# Base-Exchange Enabling the Visualization of SARM1 Activities in Sciatic Nerve-Injured Mice

**DOI:** 10.1021/acssensors.2c02317

**Published:** 2023-01-23

**Authors:** Ke Huang, Wen Jie Zhu, Wan Hua Li, Hon Cheung Lee, Yong Juan Zhao, Chi-Sing Lee

**Affiliations:** † Department of Chemistry, Hong Kong Baptist University, Waterloo Road, Kowloon Tong, Kowloon, Hong Kong SAR 999077, China; ‡ State Key Laboratory of Chemical Oncogenomics, Key Laboratory of Chemical Genomics, Peking University Shenzhen Graduate School, Shenzhen University Town, Lishui Road, Shenzhen 518055, China; § Ciechanover Institute of Precision and Regenerative Medicine, School of Life and Health Sciences, School of Medicine, The Chinese University of Hong Kong Shenzhen, Shenzhen 518172, China; ∥ School of Life Sciences, University of Science and Technology of China, Hefei, Anhui 230026, China; ⊥ Shenzhen-Hong Kong Institute of Brain Science-Shenzhen Fundamental Research Institutions, Shenzhen 518055, China

**Keywords:** base-exchange, enzyme activities, in vivo imaging, SARM1, activatable fluorescent probes

## Abstract

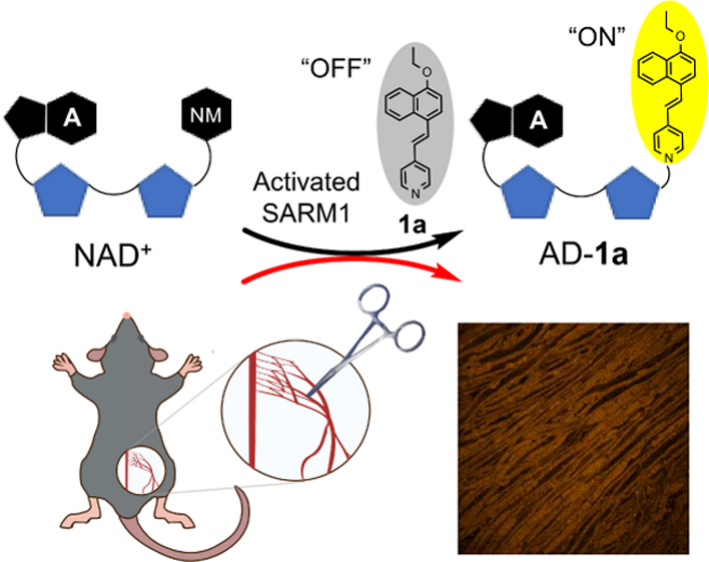

Enzymes are important in homeostasis in living organisms. Since abnormal enzyme activities are highly associated with many human diseases, detection of in vivo activities of a specific enzyme is important to study the pathology of the related diseases. In this work, we have designed and synthesized a series of new small-molecule-activatable fluorescent probes for the imaging of Sterile Alpha and TIR Motif-containing 1 (SARM1) activities based on its transglycosidase activities (base-exchange reactions of NAD^+^). Probe **1a** was found to undergo base-exchange reactions with NAD^+^ in the presence of activated SARM1 but not CD38 nor NADase and formed a highly emissive product AD-**1a** [about a 100-fold fluorescence enhancement in 20 min with a 150 nm (5665 cm^–1^) Stokes shift and a 100 nm (3812 cm^–1^) red shift]. This probe exhibited a higher reactivity and sensitivity than those commonly used for SARM1 imaging. The utilities of **1a** have also been demonstrated in live-cell imaging and detection of in vivo activities of SARM1 in a sciatic nerve injury mouse model.

## Introduction

Enzymes play a crucial role in self-regulating processes in living organisms.^1,2^ Abnormal enzyme activities could result in failure of homeostasis and lead to severe problems or even fatality.^3^ Recently, many reports have indicated that erratic up-/down-regulation of enzyme activities is closely associated with the development of many human diseases.^4−11^ Thus, detection of in vivo activities of a specific enzyme can provide valuable information for studying the pathology of the related human disease.^12^ Fluorescence imaging using small-molecule-activatable probes is a powerful tool for monitoring in vivo enzyme activities due to their ability for non-invasive visualization of a specific biological progress as well as improved sensitivity and specificity compared to the traditional activity-based “always on” fluorescent probes.^13^ Due to their promising applications in early-stage disease diagnosis and therapy efficacy monitoring, different types of activatable small-molecule fluorescence probes have been developed for monitoring the intracellular activities of a variety of enzymes.^14−16^

Our group is particularly interested in developing small-molecule-activatable fluorescent probes for in vivo imaging of Sterile Alpha and TIR Motif-containing 1 (SARM1), which is a non-redox NAD^+^-consuming enzyme that regulates axonal degeneration via its NAD-metabolizing activities^17−20^ and is a potential therapeutic target for neurodegenerative disorders and spinal cord injury.^21,22^ SARM1 is a multifunctional enzyme that can deplete cellular NAD^+^ through cyclization, hydrolysis, and transglycosidation (exchange reactions between the nicotinamide moiety of NAD^+^ and an alternative base) of NAD^+^.^23^

**Figure 1:**
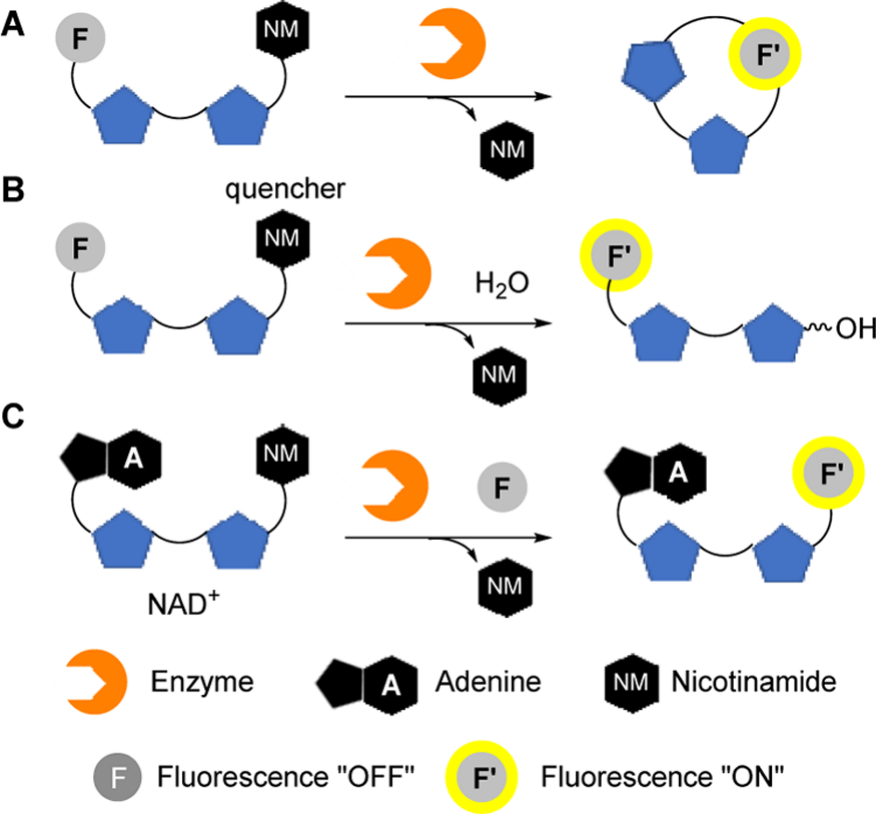
Strategies for developing activatable fluorescent probes based on (A) cyclization, (B) hydrolysis, and (C) transglycosidation of NAD^+^.

Traditionally, NAD^+^ cyclase activities can be analyzed using NAD^+^ fluorogenic enzymatic substrates,^24^ such as nicotinamide guanine dinucleotide (NGD) and nicotinamide hypoxanthine dinucleotide (NHD), which can be cyclized and form fluorescent products by the enzyme (Figure 1A). Nicotinamide 1,*N*^6^-ethenoadenine dinucleotide (εNAD) is an activatable fluorescent probe for the detection of NAD^+^ hydrolase (NADase) activities,^25^ in which the adenine ring is replaced by a pre-quenched fluorophore, 1,*N*^6^-ethenoadenine. Upon enzymatic hydrolysis of the nicotinamide (the quencher), the fluorescence of 1,*N*^6^-ethenoadenine is resumed (Figure 1B).

For imaging of the transglycosidase activities, Preugschat et al.^26^ employed a series of 4′-pyridinyl drugs such as dismerinone and pinacidil as activatable fluorescent probes for imaging the activities of CD38, a non-redox NAD-consuming enzyme found on the surface of immune cells.^27^ However, the fluorescence intensity of the base-exchanged products is much lower than that of the probe itself. Recently, our group found that a known *trans*-stilbene derivative, *trans*-4-ethoxy-4′-stilbazole,^28^ can be used as an activatable fluorescent probe for in vitro imaging of SARM1 activation, and we have demonstrated its application in the identification of a novel SARM1 inhibitor.^29^ Unfortunately, this probe is not applicable for in vivo imaging due to its moderate reactivity and fluorescence intensity of the base-exchanged products. We herein reported the development of a small-molecule-activatable fluorescent probe (**1a**), which can be utilized for in vitro and in vivo imaging of SARM1 activities.

## Results and Discussion

### Design and Synthesis of Small-Molecule Fluorescent Probes

**Figure 2:**
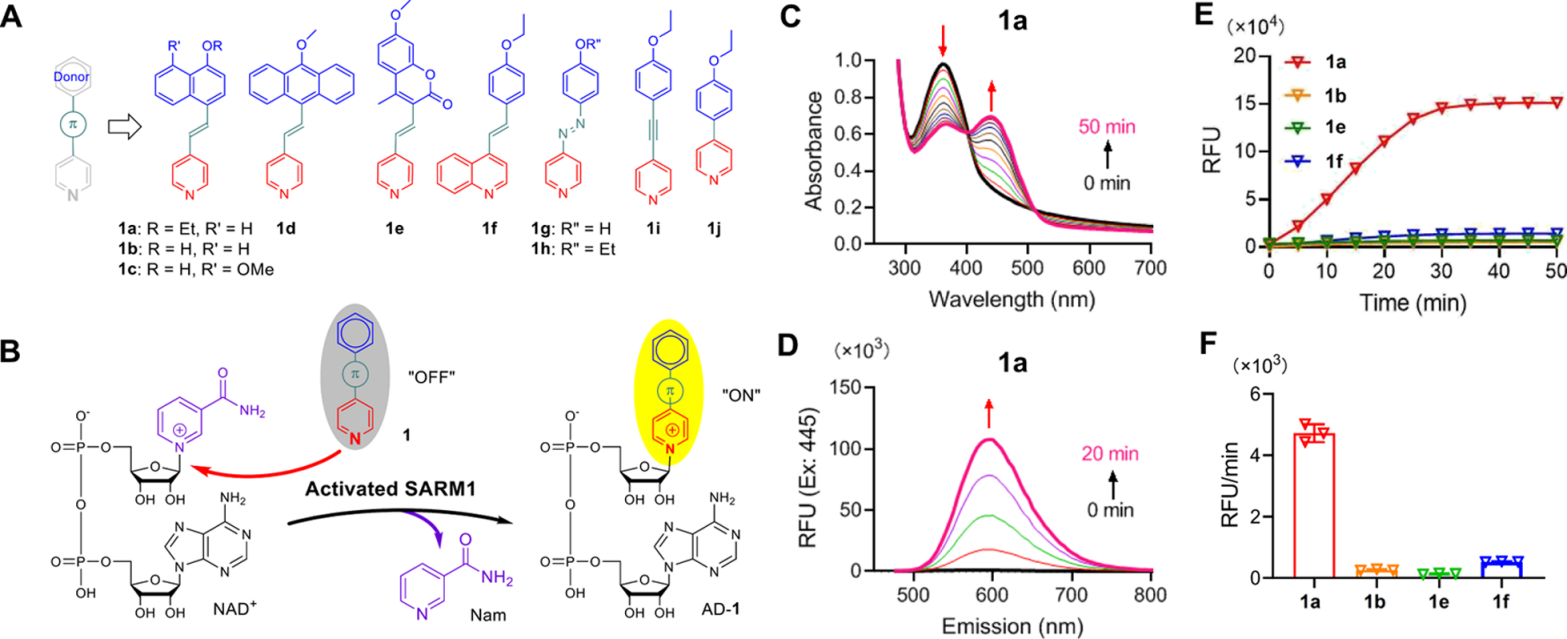
(A) Design of small-molecule-activatable fluorescent probes based on the donor−π–acceptor motif. (B) Fluorescence imaging of SARM1-dN activities with **1** via base-exchange reactions. (C) Changes in the absorbance spectra of **1a** range from 280 to 700 nm throughout time. (D) Time-dependent changes of the emission spectra of **1a** (λ_ex_ = 445 nm). (E) The fluorescence kinetics increase at the maximal absorbance wavelengths catalyzed by SARM1-dN in the presence of NMN (100 μM), NAD^+^ (100 μM), and **1** (50 μM) (**1a**/AD-**1a**: λ_ex_/λ_em_ 445/595 nm; **1b**/AD-**1b**: λ_ex_/λ_em_ 455/590 nm; **1e**/AD-**1e**: λ_ex_/λ_em_ 430/530 nm; **1f**/AD-**1f**: λ_ex_/λ_em_ 440/580 nm). (F) Reaction rate of **1** based on the fluorescence enhancement.

**Figure 3:**
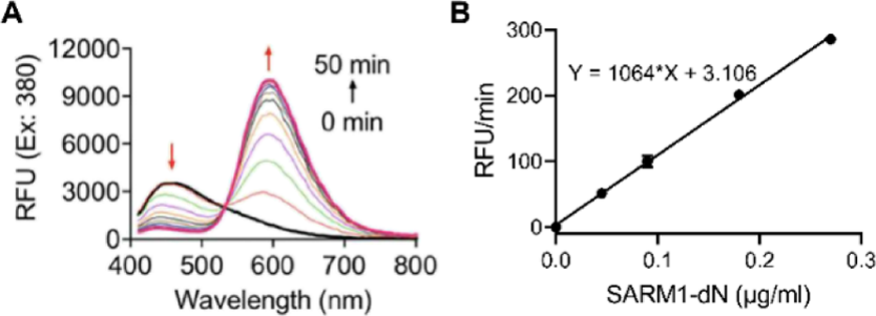
(A) Fluorescence measurement of the base-exchange reactions of **1a** at the isosbestic point (λ_ex_ = 380 nm). (B) Quantification of the reaction rate of **1a** via regression equation in the presence of gradient concentrations of SARM1-dN.

Based on the donor−π–acceptor^30^ motif of *trans*-4-ethoxy-4′-stilbazole, we have designed a series of new fluorescent probes **1a–1j** with structural modifications of the aryl donor, the π-linker, and the pyridyl acceptor (Figure 2A). These probes were synthesized readily via Pd-catalyzed cross-coupling or diazo coupling according to the standard procedures.^31^ The structures of all the fluorescent probes were fully characterized, and their photophysical properties in PBS buffer as well as the pH effects on the fluorescent changes (Figure S3) were studied. We anticipated that activated SARM1 can catalyze base-exchange reactions of NAD^+^ with **1** and lead to exchange products (AD-**1**) with a new and strong fluorescence signal (Figure 2B). To test this hypothesis, the UV absorbance and the fluorescence changes of the base-exchange reactions of **1** catalyzed by SARM1-dN (a water-soluble recombinant SARM1 with *N*-terminal mitochondrial-localizing peptide truncated) with nicotinamide mononucleotide (NMN) as the activator were investigated.^32^ Among the small-molecule probes being studied, the UV absorption maxima of **1a** (360 nm), **1b** (355 nm), **1e** (358 nm), **1f** (364 nm), **1g** (368 nm), **1h** (354 nm), and **1i** (308 nm) decrease, and new UV absorbance bands appeared at 445 nm (**1a**), 455/590 nm (**1b**), 430 nm (**1e**), 440 nm (**1f**), 550 nm (**1g**), 415 nm (**1h**), and 380 nm (**1i**), forming isosbestic points at 380, 390, 400, 390, 500, 380, and 320 nm, respectively (Figure S1, Table S1), indicating that these probes were transformed to a new species, presumably the base-exchanged products, in the presence of the NMN-activated SARM1-dN. Upon excitation at the new UV absorption bands, a new fluorescence signal was observed for **1a** (595 nm), **1b** (590 nm), **1e** (530 nm), and **1f** (580 nm), indicating a Stokes shift of 150 nm (5665 cm^–1^), 135 nm (5523 cm^–1^), 100 nm (4388 cm^–1^), and 140 nm (5486 cm^–1^), respectively (Figure S2, Table S2). To our delight, probe **1a** with the naphthalene-(*E*)-alkene-pyridine framework exhibited the largest fluorescent enhancement with about 100-fold enhancement at 595 nm in 20 min (Figure 2D–F) and a large Stokes shift at 150 nm (5665 cm^–1^) and is found to be ratiometric (Figure 3A). The large fluorescence enhancement of **1a** upon the base-exchange reaction is due to its molecular framework and the balance between the size of the π-system of the donor and the water solubility. Moreover, the fluorescence enhancement rate is proportional to the concentration of SARM1-dN with a detection limit of 6.5 ng/mL (Figure 3B). These results indicated that probe **1a** can be used as a “turn-on” fluorescent probe for the detection of SARM1 activities.

**Figure 4:**
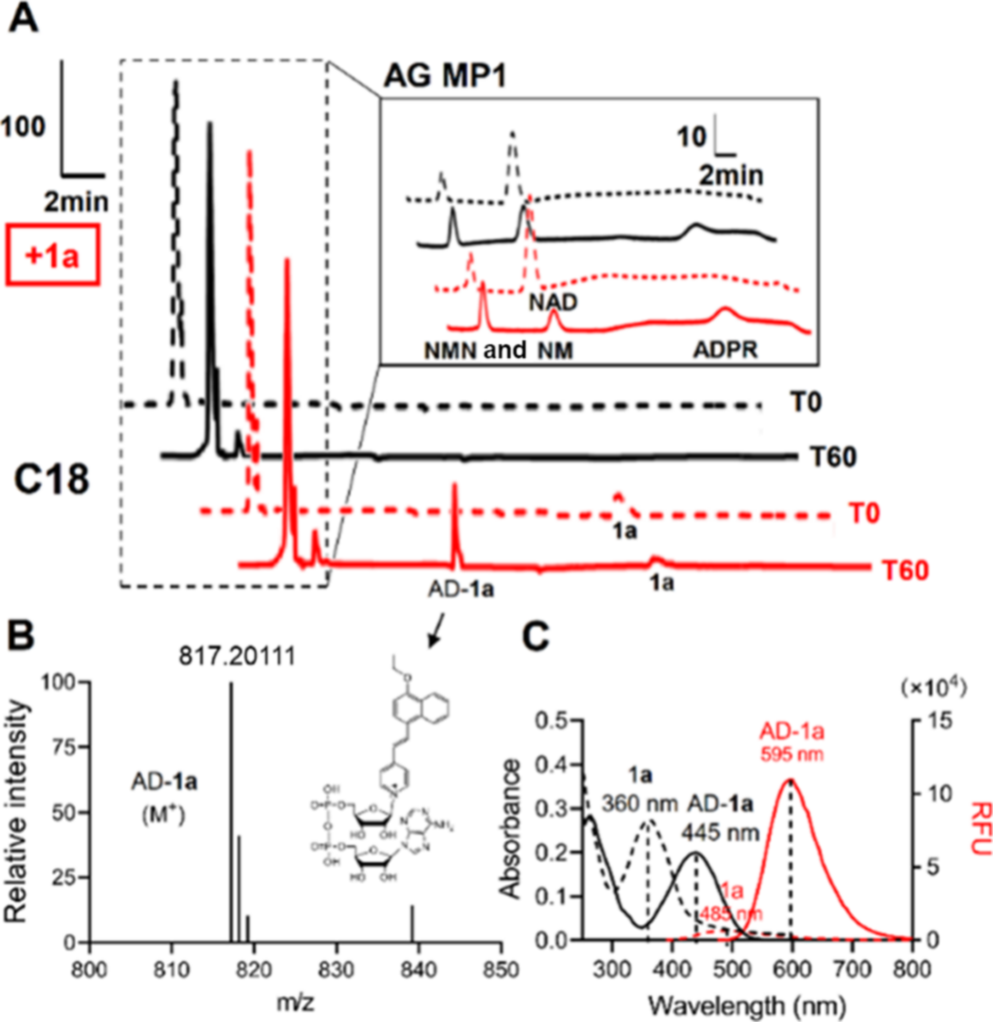
(A) HPLC analysis of the base-exchange reaction of **1a** with NAD^+^ catalyzed by SARM1-dN with NMN as the activator. (B) HRMS analysis of AD-**1a**. (C) Photophysical properties of **1a** and AD-**1a** (Stokes shift = 150 nm or 5665 cm^–1^; red shift = 100 nm or 3812 cm^–1^).

To characterize the base-exchange product AD-**1a**, the SARM1-catalyzed base-exchange reactions of **1a** were analyzed using HPLC and HRMS. In the presence of SARM1-dN with NMN as the activator, NAD^+^ was converted mainly to the hydrolyzed product, ADP-ribose (ADPR) (Figure 3A, black curve). Upon the addition of **1a**, a new peak was observed in HPLC analysis (Figure 4A, red curve), which was identified as the expected base-exchanged product (AD-**1a**) by HRMS (Figure 4B). The isolated AD-**1a** showed a UV absorption at 445 nm and fluorescence emission at 595 nm (Figure 4C), which are consistent with the UV and fluorescence signals observed in the base-exchanged reaction study. The emission of **1a** was observed at 485 nm, indicating a 100 nm (3812 cm^–1^) red shift of the fluorescence signal.

### In Vitro Studies

**Figure 5:**
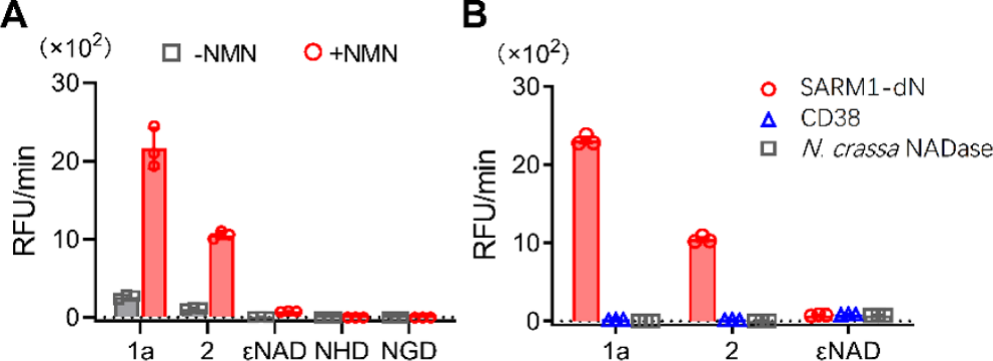
Comparison studies of (A) SARM1-dN activities with **1a** and other reported activatable fluorescent probes in PBS buffer for 50 min. (B) **1a** (10 μM) with SARM1-dN, CD38, and *N. crassa* NADase.

In an in vitro study of SARM1 activities without NMN, probe **1a** shows very weak fluorescence enhancement (Figure 5A, gray bars), indicating a slow base-exchange rate due to the autoinhibitory nature of SARM1.^33,34^ Upon the addition of NMN, probe **1a** showed a more than 100-fold higher sensitivity than those of other commonly used fluorescent probes (εNAD, NGD, and NHD) and more than double the sensitivity over our previously reported *trans*-4-ethoxy-4′-stilbazole (**2**) (Figure 5A, red bars). In addition to its high sensitivity, probe **1a** showed excellent selectivity toward SARM1 over CD38 and *Neurospora crassa* NADase.^35,36^ CD38 is also known to be able to catalyze base-exchange reactions of NAD^+^, but only SARM1 can produce a large fluorescence enhancement with **1a** (Figure 5B). Probe **2** also shows a high selectivity toward SARM1 but with a lower fluorescence increment rate, and εNAD does not show any selectivity. Based on the results of the comparison studies, probe **1a** exhibited the highest sensitivity and selectivity toward activated SARM1 among other commonly used SARM1 imaging probes.

### Live-Cell Imaging

**Figure 6:**
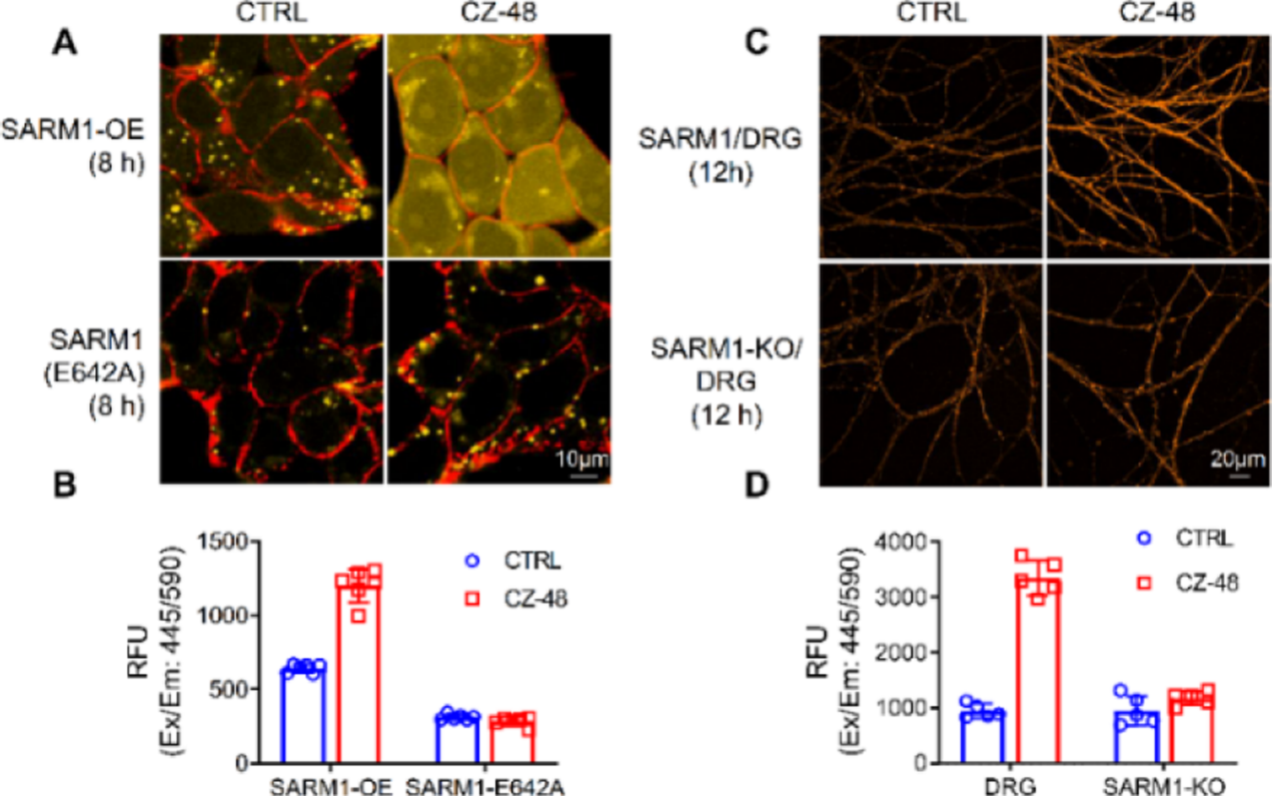
Live-cell imaging of SARM1-dN activities with **1a**. (A) Confocal fluorescence images of cells after incubation using **1a** (10 μM) with or without CZ-48 (100 μM). (B) Fluorescence intensity quantification of cells in (A). (C) Confocal fluorescence images of DRG neurons after incubation using **1a** (25 μM) with or without CZ-48 (200 μM). (D) Fluorescence intensity quantification of cells in (C). Yellow–orange: AD-**1a**; red: ConA-Alex-647.

With its remarkable results in hand, probe **1a** was employed for live-cell imaging. As shown in Figure 6A, only faint yellow–orange fluorescence was observed in HEK cells with overexpressing wildtype SARM1 after 8 h of incubation. In the presence of a cell-permeant NMN mimetic (CZ-48),^32^ the yellow–orange fluorescence enhanced very strongly inside the cells after 8 h of incubation, indicating the activation of SARM1 by CZ-48 and the cellular impermeability of the base-exchanged product AD-**1a**. On the other hand, no yellow–orange fluorescence was observed with the enzymatically inactive mutant (E642A) with or without CZ-48. Live-cell imaging was also studied using mouse dorsal root ganglion (DRG) neurons (Figure 6C). The yellow–orange fluorescence of AD-**1a** can be observed clearly with CZ-48 as the activator, while the SARM1-knockout DRG neurons have no significant difference with or without CZ-48.

### In Vivo Studies

**Figure 7:**
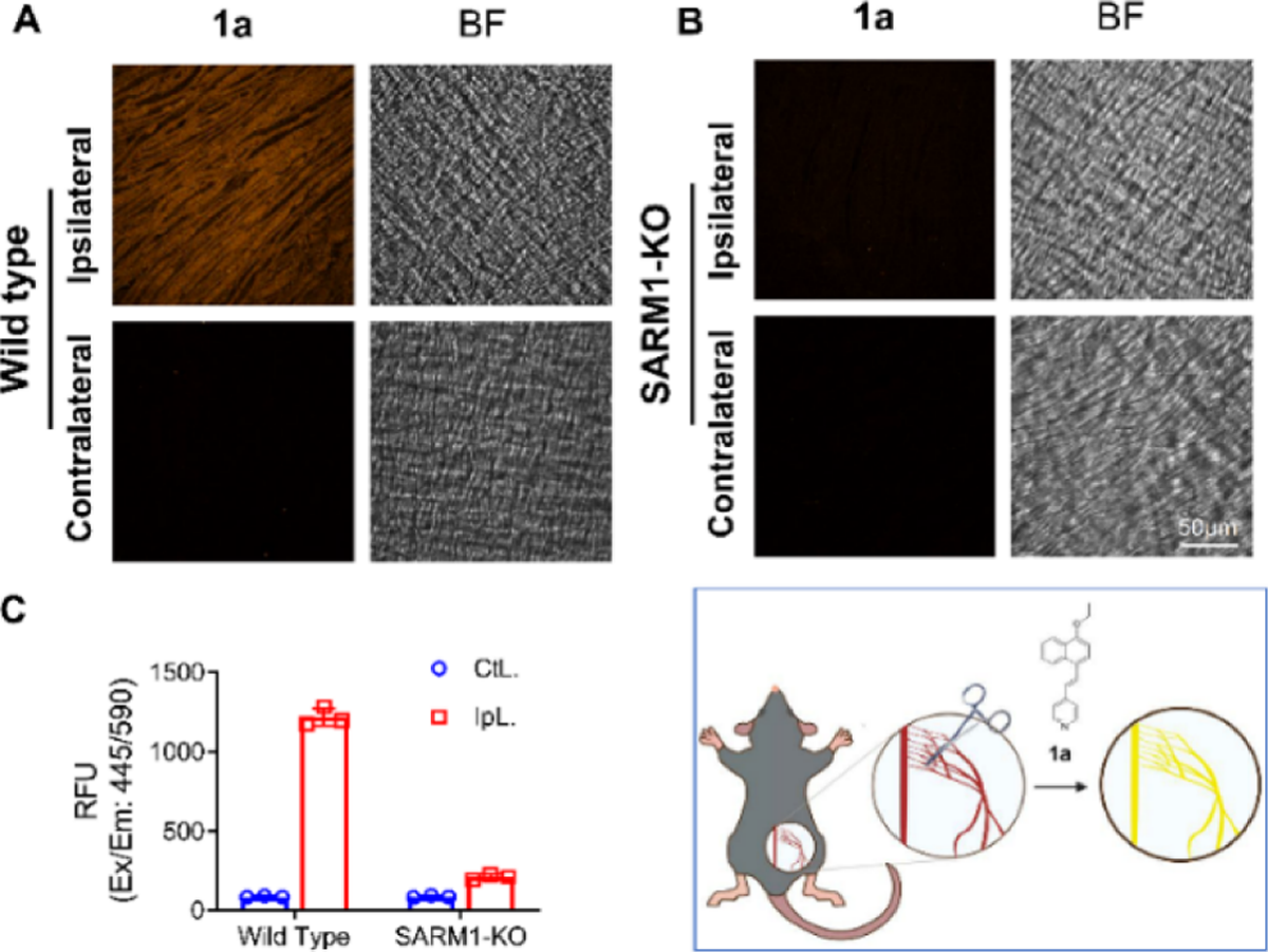
In vivo imaging of SARM1 activities by **1a**. (A) Confocal fluorescence images of the sciatic nerve after incubation with **1a** (1 mg/mL) in the injured nerve of wildtype and (B) SARM1-KO mice. (C) Fluorescent intensity quantification of the sciatic nerve in (A). Yellow orange: AD-**1a** (λ_ex_ = 445 nm; λ_em_ = 595 nm); BF: bright field.

In a sciatic nerve injury mouse model,^37^ the sciatic nerves in the right legs of both wildtype and SARM1-KO mice were sheared off but not that on the contralateral side. After incubation with **1a**, the yellow–orange fluorescence signals of the injured sciatic nerve on the ipsilateral side can be observed clearly but not on the non-injured sciatic nerve on the contralateral side in wildtype mice (Figure 7A). This result indicates that SARM1 is activated in the injured sciatic nerves but not in the non-injured sciatic nerves. On the other hand, no yellow–orange fluorescence signal is observed in both injured and non-injured sciatic nerves in SARM1-OK mice (Figure 7B). The results of this animal study demonstrated that probe **1a** can be used for fluorescence imaging of in vivo activities of SARM1 in injury-induced axonal degeneration. To the best of our knowledge, this is the first example of in vivo imaging of SARM1 activities using a small-molecule-activatable fluorescent probe.

## Conclusions

In conclusion, we developed a novel small-molecule-activatable fluorescent probe **1a** for in vitro and in vivo imaging of SARM1. Probe **1a** undergoes base-exchange reactions with NAD^+^ in the presence of activated SARM1-dN and forms a highly emissive cell-impermeable base-exchanged product AD-**1a**, which exhibits a 100-fold fluorescence enhancement in 20 min with a large Stokes (150 nm or 5665 cm^–1^) and red shift (100 nm or 3812 cm^–1^). In comparison studies, this probe exhibited nigher reactivity and sensitivity than those commonly used for SARM1 imaging. This probe was successfully utilized in live-cell fluorescence imaging of SARM1 activities in HEK SARM1-OE and DRG neuron cells. More importantly, it was demonstrated as the first small-molecule-activatable fluorescent probe for in vivo imaging of SARM1 activities in a sciatic nerve injury mouse model. This probe would find potential applications in identifying novel SARM1 inhibitors and studying the pathology of related neurodegenerative disorders.

## Experimental Section

### Synthesis of **1a**

To a stirred solution of 4-bromonaphthalen-1-ol (0.60 g, 3.0 mmol) in DMF (7 mL) were added ethyl bromide (0.27 mL, 5.0 mmol) and K_2_CO_3_ (1.0 g, 9.4 mmol) at rt. The resulting mixture was stirred at 70 °C for 6 h. The mixture was then diluted with water (20 mL), and the aqueous phase was extracted with ethyl acetate (15 mL ×3). The combined organic extracts were washed with water (15 mL ×3), dried over anhydrous Na_2_SO_4_, filtered, and evaporated under reduced pressure. To a stirred solution of the resulting white solid (crude), 4-vinylpyridine (316 mg, 3.0 mmol), P(*o*-tol)_3_ (183 mg, 20 mol %), and triethylamine (1.2 mL, 8.7 mmol) in degaussed CH_3_CN under argon was added Pd(OAc)_2_ (69 mg, 10 mol %) quickly. The resulting mixture was stirred at 100 °C for 5 h. The mixture was then cooled to rt and diluted with water (30 mL). The aqueous phase was extracted with ethyl acetate (15 mL ×3). The combined organic extracts were dried over anhydrous Na_2_SO_4_, filtered, and evaporated under reduced pressure. Silica gel flash column chromatography (ethyl acetate/hexanes = 3:1) of the residue gave a pale orange solid (232 mg, 28% yield) as the product. **1a**: mp = 130–131 °C. ^1^H NMR (400 MHz, chloroform-*d*): δ 8.65 (dd, *J* = 4.6, 1.6 Hz, 2H), 8.43 (dd, *J* = 8.3, 1.0 Hz, 1H), 8.20 (d, *J* = 8.2 Hz, 1H), 8.09 (d, *J* = 16.0 Hz, 1H), 7.76 (d, *J* = 8.1 Hz, 1H), 7.61 (dddd, *J* = 25.0, 8.1, 6.8, 1.3 Hz, 2H), 7.49 (dd, *J* = 4.7, 1.5 Hz,2H), 7.03 (d, *J* = 16.0 Hz, 1H), 6.91 (d, *J* = 8.1 Hz, 1H), 4.31 (q, *J* = 7.0 Hz, 2H), 1.64 (t, *J* = 7.0 Hz, 3H). ^13^C NMR (100 MHz, chloroform-*d*): δ 155.4, 150.0, 145.0, 132.0, 130.0, 126.8, 126.4, 125.7, 125.5, 125.1, 124.5, 122.9, 122.6, 120.6, 104.4, 63.6, 14.6. HRMS (+ESI) *m*/*z* calcd for C_19_H_17_NO (M + H)^+^, 276.1383; found, 276.1382.

### Preparation and Quantification of the Enzymes

The preparation of the SARM1 truncated form, SARM1-dN, has been described previously.^29^ SARM1 was expressed in HEK293T cells lacking the N-terminal mitochondrial signal and released by digitonin (100 μM) in PBS with the protease inhibitor cocktail (Roche). The negative control was a cell lysate from wildtype HEK293T produced using the same procedure. To measure SARM1-dN, BC2 nanobody-conjugated (Bruce and McNaughton, 2017) beads were used, which were made by combining the BC2 nanobody with NHS-beads (GE Healthcare). The pure SARM1-dN, named SARM1-IP, was run on SDS-PAGE with a little quantity of standard protein BSA, which was stained with Coomassie blue. The protein content of SARM1-dN was then quantified using the band intensity with standard BSA.

DtSARM1-dN was cloned into pLenti-CMV-puro-Dest (Invitrogen) using the LR Clonase II enzyme, with the N-terminal targeting signal deleted and tagged with a tandem strep tag II and flag tag for purification. Lentivirus infection was used to create HEK293F cells over-expressing dtSARM1-dN, which were then chosen with puromycin (1 μg/mL). DtSARM1-dN was immunoprecipitated using StrepTactin resin (GE Healthcare), washed four times with buffer W [Tris-HCl (100 mM, pH 8.0), NaCl (150 mM), and EDTA (1 mM)], and eluted with biotin (2 mM) in buffer W.

The preparation of recombinant CD38 and *N. crassa* NADase has been described previously.^36,38^

### Fluorescence Assays In Vitro

To test the SARM1 activity with **1** in vitro, reactions were initiated by incubating the enzyme with the reaction mixture, which included **1** (50 μM), NAD^+^ (100 μM), and NMN (100 μM) in PBS for 50 min. In an Infinite M200 PRO microplate reader (Tecan), absorbance and fluorescence were measured in a quartz cuvette or black 96-well plates (Corning), respectively.

### HPLC Analysis

The samples for the HPLC analysis were prepared by mixing SARM1 (MBP-dtSARM1 purified by MBP-beads, around 5 μg/mL) with NAD^+^ (100 μM), **1a** (50 μM), NMN (100 μM), and BSA in PBS (0.1 mg/mL) and incubated at 37 °C for 60 min. The mixtures were applied to a C-18 reverse phase column equipped on a high-performance liquid chromatograph (Agilent 1260) with a gradient of CH3CN from 30 to 70% to elute AD-**1a** and **1a**. The hydrophilic peaks of the C-18 reverse-phase column were collected and applied to an AG-MP1 column equipped on a high-performance liquid chromatograph (Agilent 1260) with a gradient of 100 mM trifluoroacetic acid from 5 to 40% to elute to NM, NMN, ADPR, and NAD. The AD-**1a** fractions were collected and lyophilized for the characterization of absorption, fluorescence spectra, and mass spectrometry.

### Confocal Imaging in Living Cells

HEK293 cells overexpressing wildtype or the enzymatically dead form (E642A) of SARM1 or HEK293T cells knocking out NMNAT1 have been created previously.^5^ Cells were cultured overnight on 0.05 mg/mL poly-l-lysine-coated chambered coverglass (Thermo Fisher, #155411) and then treated with **1a** (10 μM) in the presence or absence of CZ-48 (100 μM) for 8 h (for SARM1-OE and E642A cells). Before imaging, the cells were stained with concanavalin A (50 μg/mL) and Alexa Fluor 647 Conjugate (Thermo Fisher) at 4 °C for 10 min to show the cell edges. A confocal microscope was used to acquire the fluorescent signals (λ_ex_/λ_em_: 445/595 nm for **1a**; λ_ex_/λ_em_: 561/590 nm for ConA) (Nikon A1).

### DRG Culture and Imaging

The DRG culture in mice was carried out as reported.^39^ DRGs were dissected from embryos at days 12.5 to 14.5 (E12.5–E14.5), dispersed in 0.05% trypsin solution containing 0.02% EDTA (Gibco), and seeded in Neurobasal Plus Medium supplemented with 2% B27 plus, GlutaMax (1 mM), 1% penicillin/streptavidin solution, 5-fluoro-2′-deoxyuridine (1 μM), uridine (1 μM), and NGF (37.5 ng/mL) on a chambered coverglass pre-coated with poly-l-lysine (0.1 mg/mL), laminin (0.02 mg/mL), and 5% FBS. Half of the culture media was changed every 3 days with new media.

The neurons on Div6-8 were treated with **1a** (25 μM) in the absence or presence of 200 μM CZ-48 for 12 h. A confocal microscope (Nikon A1) with a 60× object was used to collect the fluorescence images (λ_ex_/λ_em_: 445/595 nm for AD-**1a**). NIS-Elements AR analysis was used to determine the mean fluorescence intensity (Nikon A1).

### Animals

C57BL6/J mice were purchased from the Guangdong Medical Laboratory Animal Center (China). SARM1-KO (JAX: 018069) mice were from The Jackson Laboratory (stock no: 018069) and were donated from Zeng Wenwen’s group (Tsinghua University). The mice were housed under a 12 h day/night cycle at 25.0 °C with a standard diet and clean water. All animal experiments were approved by the Peking University Shenzhen Graduate School Animal Care and Use Committee (#AP0015003).

### Confocal Imaging of Mice

6–8 week old C57BL/6 wildtype and SARM1-KO mice were anesthetized with pentobarbital sodium (50 mg/kg) via intraperitoneal injection. Using scissors and forceps, the sciatic nerve was found in the right leg and sheared off. The same operation was conducted on the contralateral side without shearing the nerve off. Both operation sides were kept with 200 μL 1 mg/mL **1a** (3.5% Tween 80, 5% dimethylacetamide, 20% PEG400, 20% propylene glycol, 35% PBS, and 16.5% water) for 12 h, while the mice were kept on the warm operating table with 0.5% isoflurane gas anesthesia. The sciatic nerve was isolated immediately, and a confocal microscope was used to acquire the fluorescent signals (λ_ex_/λ_em_: 445/595 nm for **1a**) (Nikon A1).

## Supporting Information Available

Synthesis, characterization data, and NMR spectra of fluorescent probes and UV absorption and fluorescence spectra of exchange reactions of **1** catalyzed by activated SARM1-dN (PDF)
