# Supplementary material for: Base-Exchange Enabling the Visualization of SARM1 Activities in Sciatic Nerve-Injured Mice
Source: ACS Sens. 2023 Jan 23;8(2):767–73. doi: 10.1021/acssensors.2c02317 (PMC9972468; doi:10.1021/acssensors.2c02317)
Supplement: Supplementary file 1 — se2c02317_si_001.pdf [file se2c02317_si_001.pdf]

Supporting Information

Base-exchange Enabling Visualization of SARM1 Activities in Sciatic Nerve-injured Mice

Ke Huang,[1†] Wen Jie Zhu,[2†] Wan Hua Li,[2,3,4†] Hon Cheung Lee,[2*] Yong Juan Zhao,[2,3,5*] and Chi-Sing Lee[1*]

[1]*Department of Chemistry, Hong Kong Baptist University, Waterloo Road, Kowloon Tong, Hong Kong SAR, China.*

[2]*State Key Laboratory of Chemical Oncogenomics, Key Laboratory of Chemical Genomics, Peking University Shenzhen Graduate School, Shenzhen University Town, Lishui Road, Shenzhen 518055, China.*

[3]*Ciechanover Institute of Precision and Regenerative Medicine, School of Life and Health Sciences, School of Medicine, The Chinese University of Hong Kong Shenzhen, Shenzhen, 518172, China.*

[4]*School of Life Sciences, University of Science and Technology of China, Hefei, Anhui 230026, China.*

[5]*Shenzhen-Hong Kong Institute of Brain Science-Shenzhen Fundamental Research Institutions, Shenzhen 518055, China.*

[†]*These authors contributed equally to this work.*

Table of Contents

1. Synthesis of new compounds……………………………………..……………………..…S2 – S12

2. NMR spectra of new compounds…………………………..……………………………S13 – S22

3. Supplementary Figures and Tables…………………………..………………………….S23 – S25

4. References……………………………….…………………………………………………..S25

1. Synthesis of new compounds

Materials and reagents for chemical synthesis

All the reactions which are sensitive to air and water were processed with anhydrous solvents in pre-dried flasks under the protection of an argon atmosphere, unless otherwise specified. All the reactants were purchased commercially and used directly with no further purification, unless otherwise specified. Anhydrous DMF was vacuum distilled from barium oxide. Anhydrous acetonitrile and dichloromethane were purchased commercially. All the reactions were monitored by thin-layer chromatography (TLC) processed on 0.25 mm silica gel plates (60F-254), which were analyzed by UV light at 254 nm and 365 nm and by staining with anisaldehyde, iodine-stain method or $KMnO_4$ solutions. Purification of the intermediates and products was carried out on Silica gel (200-300 mesh) by flash column chromatography. All new compounds were characterized by ^{1}H NMR, ^{13}C NMR and HRMS.

Synthesis of 1a

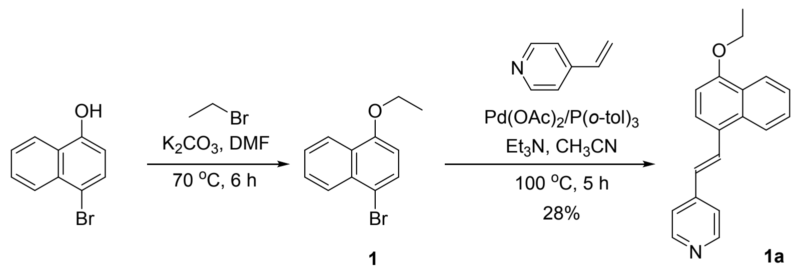

To a stirred solution of 4-bromonaphthalen-1-ol (0.60 g, 3.0 mmol) in DMF (7 mL) was added ethyl bromide (0.27 mL, 5.0 mmol), and K_2CO_3 (1.0 g, 9.4 mmol) at rt. The resulting mixture was stirred at 70 °C for 6 h. The mixture was then diluted with water (20 mL), and the aqueous phase was extracted with ethyl acetate (15 mL × 3). The combined organic extracts were washed with water (15 mL × 3), dried over anhydrous Na_2SO_4, filtered and evaporated under reduced pressure. A white solid was obtained as intermediate **1**. The crude intermediate was used directly in next step without further purification.

To a stirred solution of the above crude intermediate **1**, 4-vinylpyridine (316 mg, 3.0 mmol), P(*o*-tol)$_3$ (183 mg, 20 mol%) and triethylamine (1.2 mL, 8.7 mmol) in degassed CH_3CN (30 mL) under argon was added $Pd(OAc)_2$ (69 mg, 10 mol%) quickly. The resulting mixture was stirred at 100 °C

for 5 h. The mixture was then diluted with water (30 mL) and the aqueous phase was extracted with ethyl acetate (15 mL × 3). The combined organic extracts were dried over anhydrous Na_2SO_4, filtered and evaporated under reduced pressure. Silica gel flash column chromatography (EtOAc / hexanes = 3:1) of the residue gave a pale orange solid (232 mg, 28% yield) as the product. **1a**: mp = 130-131 °C. ^{1}H NMR (400 MHz, chloroform-*d*) δ 8.65 (dd, *J* = 4.6, 1.6 Hz, 2H), 8.43 (dd, *J* = 8.3, 1.0 Hz, 1H), 8.20 (d, *J* = 8.2 Hz, 1H), 8.09 (d, *J* = 16.0 Hz, 1H), 7.76 (d, *J* = 8.1 Hz, 1H), 7.61 (dddd, *J* = 25.0, 8.1, 6.8, 1.3 Hz, 2H), 7.49 (dd, *J* = 4.7, 1.5 Hz,2H), 7.03 (d, *J* = 16.0 Hz, 1H), 6.91 (d, *J* = 8.1 Hz, 1H), 4.31 (q, *J* = 7.0 Hz, 2H), 1.64 (t, *J* = 7.0 Hz, 3H). ^{13}C NMR (100 MHz, Chloroform-*d*) δ 155.4, 150.0, 145.0, 132.0, 130.0, 126.8, 126.4, 125.7, 125.5, 125.1, 124.5, 122.9, 122.6, 120.6, 104.4, 63.6, 14.6. HRMS(+ESI) m/z calcd. For $C_{19}H_{17}NO$ $(M+H)^+$ 276.1383, found 276.1382.

Synthesis of 1b

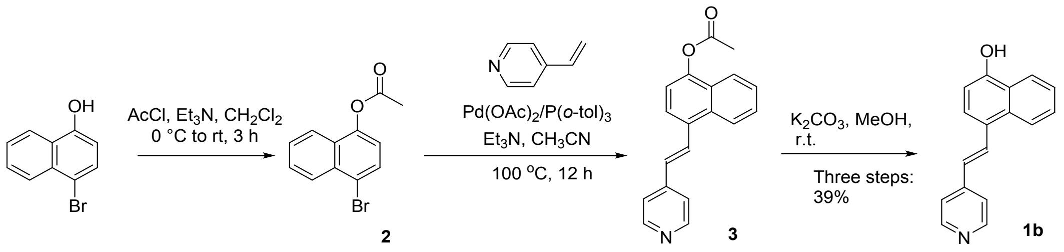

To a stirred solution of 4-bromo-1-naphthol (1.1 g, 5 mmol) and triethylamine (750 mg, 7.4 mmol) in CH_2Cl_2 (20 mL) was added acetyl chloride (465 mg, 5.92 mmol) at rt. The resulting mixture was stirred at 0 °C for 30 min and then rt for 2.5 h. The solution was then diluted with water (20 mL) and the aqueous phase was extracted with ethyl acetate (15 mL × 3). The combined extracts were dried over anhydrous Na_2SO_4, filtered and evaporated under reduced pressure. The resulting creamy solid was obtained as the crude intermediate **2** and was used for the next step without any further purification.

To a stirred solution of the above crude intermediate **2**, 4-vinylpyridine (525.6 mg, 5.0 mmol), P(*o*-tol)$_3$ (305 mg, 20 mol%) and triethylamine (2 mL, 15 mmol) in degassed CH_3CN (30 mL) under argon was added Pd(OAc)$_2$ (112 mg, 10 mol%) quickly. The resulting mixture was stirred at 100 °C for 12 h. The mixture was then directly poured into ice water, and the product was

precipitated, filtered. The crude was washed with hexanes three times and dissolved by EA. Then the organic solvent was evaporated under reduce pressure to give crude intermediate **3** (556.5 mg).

To the stirred solution of the above crude intermediate **3** (556.5 mg) in MeOH (5 mL) was added K_2CO_3 (798 mg, 5.8 mmol). The reaction was stirred for 2 h. The MeOH was evaporated under reduced pressure, and then diluted with water (10 mL) and the aqueous phase was extracted with ethyl acetate (15 mL × 3). The combined organic extracts were dried over anhydrous Na_2SO_4, filtered and evaporated under reduced pressure. Silica gel flash column chromatography (EtOAc / hexanes = 1:1) of the residue gave an orange solid (475.4 mg, 36% yield) as the product **1b**. mp = 207.9-208.0 °C. ^{1}H NMR (400 MHz, DMSO-d_6) δ 10.50 (s, 1H), 8.59 – 8.45 (m, 2H), 8.37 (d, J = 8.5 Hz, 1H), 8.29 – 8.16 (m, 2H), 7.80 (d, J = 8.0 Hz, 1H), 7.67 (d, J = 6.2 Hz, 2H), 7.57 (ddd, J = 8.4, 6.7, 1.5 Hz, 1H), 7.49 (ddd, J = 8.1, 6.8, 1.2 Hz, 1H), 7.11 (d, J = 16.0 Hz, 1H), 6.93 (d, J = 8.0 Hz, 1H). ^{13}C NMR (101 MHz, DMSO-d_6) δ 154.3, 149.9, 144.8, 132.1, 129.6, 126.7, 125.4, 124.8, 124.7, 124.4, 123.9, 123.5, 122.5, 120.9, 108.3. HRMS (+MALDI-TOF) m/z calcd. for $C_{17}H_{13}NO$ $(M+H)^+$ 248.1069, found 248.1057.

Synthesis of 1c

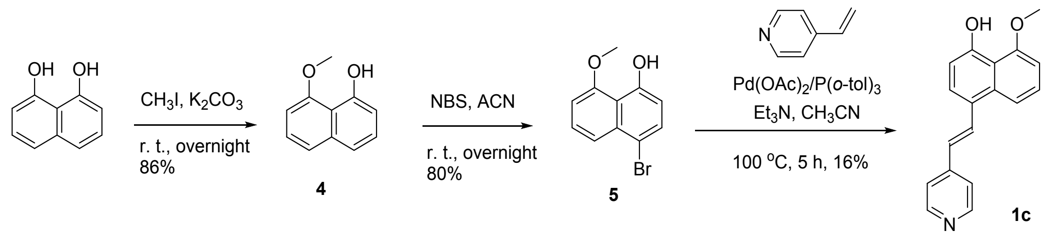

1,8-Naphthalenediol (1.6 g, 10.0 mmol) was dissolved in 50 mL of acetone, and K_2CO_3 (1.72 g, 12.5 mmol) and CH_3I (0.93 mL, 15 mmol) were added sequentially at rt. The resulting heterogeneous mixture was stirred at this temperature for 24 h. It was then quenched with H_2O (5 mL) and saturated with NH_4Cl solution (50 mL). Acetone was evaporated under reduced pressure. The aqueous phase was extracted with EtOAc (2 × 10 mL). The combined organic phase was dried over anhydrous Na_2SO_4, filtered, and concentrated under reduced pressure. Purification by flash column chromatography (EtOAc / hexane = 1:20) gave **4** (1.49 mg, 86% yield) as a white solid. Spectral data of **4** is consistent with those reported in the literature.[1]

To a stirred solution of **4** (522.5 mg, 3 mmol) in ACN (12 mL) was added NBS (533.9 mg, 3 mmol). The resulting dark solution was stirred overnight, concentrated, and purified by flash column chromatography (EtOAc / hexane = 1:20) to give **5** as a white solid (604.1 mg, 80% yield). Spectral data of **5** is consistent with those reported in the literature.[1]

To a stirred solution of **5** (506.2 mg, 2 mmol), 4-vinylpyridine (210 mg, 2.0 mmol), P(*o*-tol)$_3$ (122 mg, 20 mol%) and triethylamine (0.83 mL, 6 mmol) in degassed CH_3CN (20 mL) under argon was added Pd(OAc)$_2$ (45 mg, 10 mol%) quickly. The resulting mixture was stirred at 100 °C for 5 h. The mixture was then diluted with water (30 mL) and the aqueous phase was extracted with ethyl acetate (15 mL × 3). The combined organic extracts were dried over anhydrous Na_2SO_4, filtered and evaporated under reduced pressure. Silica gel flash column chromatography (EA / hexanes = 1:1) of the residue gave an orange solid (88.4 mg, 16% yield) as the product **1c**. mp = 170.3-170.4 °C. ^{1}H NMR (400 MHz, DMSO-d_6) δ 9.74 (s, 1H), 8.52 (d, J = 6.1 Hz, 2H), 8.17 (d, J = 16.0 Hz, 1H), 8.00 – 7.91 (m, 1H), 7.81 (d, J = 8.2 Hz, 1H), 7.69 – 7.60 (m, 2H), 7.47 (dd, J = 8.6, 7.7 Hz, 1H), 7.10 (d, J = 16.0 Hz, 1H), 7.03 (d, J = 7.6 Hz, 1H), 6.85 (d, J = 8.2 Hz, 1H), 4.03 (s, 3H). ^{13}C NMR (101 MHz, Chloroform-d) δ 156.3, 151.0, 145.8, 131.0, 128.0, 127.2, 127.1, 122.0, 118.1, 115.6, 111.6, 106.0, 57.4. HRMS (+MALDI-TOF) m/z calcd. for $C_{18}H_{15}NO_2$ $(M+H)^+$ 278.1176 found 278.1169.

Synthesis of 1d

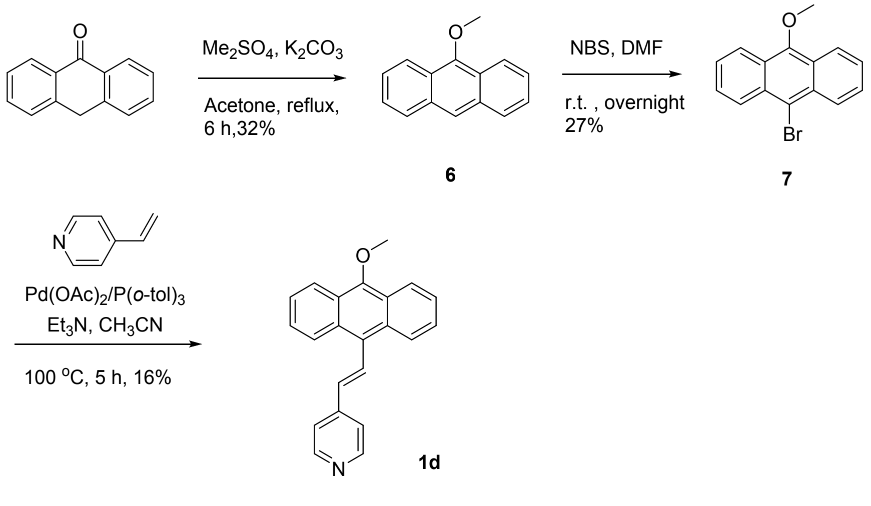

A solution of 10H-Anthracen-9-one (0.97 g, 5 mmol) and potassium carbonate (3.0 g, 21.8 mmol) in acetone (10 mL) was prepared in 50 mL round-bottomed flask under argon atmosphere.

Dimethyl sulfate (1 mL, 10.5 mmol) was added to the solution, and the reaction mixture was refluxed for 6 h. After cooling, water was added to the mixture and the product was extracted with a mixture of hexane and dichloromethane. The combined organic layer was washed with water and brine, dried over anhydrous Na_2SO_4, and filtrated. The solvent was removed in vacuo. Silica gel flash column chromatography (EtOAc / hexanes = 1:1) of the residue gave **6** (331.4 mg, 32%) as a pale-yellow crystal. Spectral data of **6** is consistent with those reported in the literature.[2]

A solution of **6** (208 g, 1 mmol) in DMF (1.4 mL) was prepared in 25-mL round-bottomed flask under argon atmosphere and cooled on ice-bath. To this solution, NBS (198.7 g, 1.12 mmol) in DMF (1.4 mL) was added dropwise via an additional funnel for 30 min, and the mixture was stirred overnight. Then, the reaction mixture was washed with water and extracted with dichloromethane. After drying over anhydrous Na_2SO_4, the solvent was removed in vacuo, and column chromatography on silica gel (hexane / EtOAc = 100:1) afforded the pale-yellow solid (77.1 mg, 27% yield) as the intermediate **7**. Spectral data of **7** is consistent with those reported in the literature.[2]

To a stirred solution of **7**, 4-vinylpyridine (116 mg, 0.033 mmol), P(*o*-tol)$_3$ (18.3 mg, 20 mol%) and triethylamine (0.1 mL, 0.08 mmol) in degassed CH_3CN (5 mL) under argon was added $Pd(OAc)_2$ (7 mg, 10 mol%) quickly. The resulting mixture was stirred at 100 °C for 5 h. The mixture was then diluted with water (10 mL) and the aqueous phase was extracted with ethyl acetate (10 mL × 3). The combined organic extracts were dried over anhydrous Na2SO4, filtered and evaporated under reduced pressure. Silica gel flash column chromatography (EA / hexanes = 1:1) of the residue gave an orange solid (88.4 mg, 16% yield) as the product **1d**. mp = 109.7-109.8. ^{1}H NMR (400 MHz, chloroform-*d*) δ 8.68 (d, *J* = 5.1 Hz, 2H), 8.42 – 8.33 (m, 2H), 8.32 – 8.27 (m, 2H), 8.13 (d, *J* = 16.5 Hz, 1H), 7.52 (ddt, *J* = 7.9, 6.8, 3.3 Hz, 6H), 6.89 (d, *J* = 16.5 Hz, 1H), 4.18 (s, 3H).^{13}C NMR (101 MHz, chloroform-*d*) δ 156.7, 155.8, 150.2, 145.2, 134.4, 130.6, 126.8, 126.6, 126.4, 124.8, 120.8, 117.5, 115.1, 110.8, 104.4, 56.3. HRMS (+MALDI-TOF) m/z calcd. for $C_{22}H_{17}NO$ $(M+H)^+$ 312.1382, found 312.1388.

Synthesis of 1e

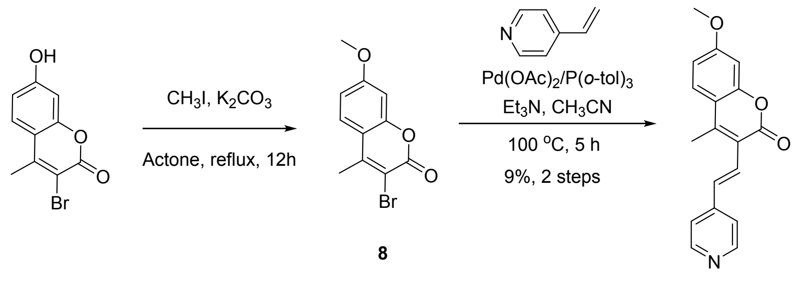

3-Bromo-7-hydroxy-4-methylchromen-2-one (3 g, 11.8 mmol), iodomethane (2.2 mL, 35.28 mmol), K_2CO_3 (2.5 g, 17.64 mmol) was add in 50mL acetone. Then the reaction was refluxed for 12h. The solvent was evaporated under reduced pressure to give a white solid (**8**). The crude intermediate **8** was used directly in next step without further purification.

To a stirred solution of the above crude intermediate **8**, 4-vinylpyridine (1.24 g, 11.8 mmol), P(*o*-tol)$_3$ (718 mg, 20 mol%) and triethylamine (4.9 mL, 35.4 mmol) in degassed CH_3CN (50 mL) under argon was added Pd(OAc)$_2$ (69 mg, 10 mol%) quickly. The resulting mixture was stirred at 100 °C for 5 h. The mixture was then diluted with water (50 mL) and the aqueous phase was extracted with ethyl acetate (40 mL × 3). The combined organic extracts were dried over anhydrous Na_2SO_4, filtered and evaporated under reduced pressure. Silica gel flash column chromatography (EtOAc / hexanes = 2:1) of the residue gave a pale-yellow solid (297 mg, 9% yield) as the product **1e**. mp = 189.7-189.9. ^{1}H NMR (400 MHz, chloroform-*d*) δ 8.69 – 8.50 (m, 2H), 7.75 – 7.63 (m, 2H), 7.45 – 7.32 (m, 3H), 6.99 – 6.79 (m, 2H), 3.93 (s, 3H), 2.63 (s, 3H). ^{13}C NMR (101 MHz, Chloroform-*d*) δ 162.7, 159.9, 154.0, 150.2, 148.5, 145.2, 132.6, 126.3, 125.1, 121.0, 118.0, 114.0, 112.9, 100.4, 55.8, 15.4. HRMS (+MALDI-TOF) m/z calcd. for $C_{18}H_{15}NO_3$ $(M+H)^+$ 294.1125, found 255.1119.

Synthesis of 1f

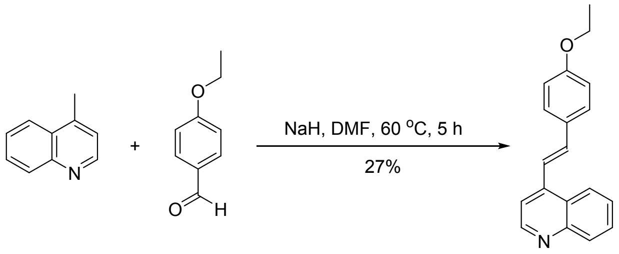

To a stirred solution of 4-methylquinoline (143 mg, 1.0 mmol) in anhydrous DMF (0.8 mL) was added NaH (52 mg of a 60% dispersion in mineral oil, 1.3 mmol) at rt. The resulting mixture was stirred at 60 °C for 2 h and then treated with 4-ethoxybenzaldehyde (225 mg, 1.5 mmol). The resulting mixture was stirred at 60 °C for 5 h. After cooling to rt, the mixture was poured into MeOH (20 mL) and diluted with water (20 mL). The aqueous phase was extracted with ethyl acetate (15 mL × 3) and the combined organic extracts were dried over anhydrous Na_2SO_4, filtered and evaporated under reduced pressure. Silica gel flash column chromatography (EtOAc / hexanes = 3:1) gave a pale-yellow solid (73 mg, 27% yield) as the product. **1f**: mp = 79-80 °C ^{1}H NMR (400 MHz, chloroform-*d*) δ 8.93 (d, J = 4.6 Hz, 1H), 8.31 – 8.23 (m, 1H), 8.22 – 8.13 (m, 1H), 7.81 – 7.56 (m, 6H), 7.34 (d, J = 15.7 Hz, 1H), 6.99 (d, J = 8.7 Hz, 2H), 4.13 (q, J = 7.0 Hz, 2H), 1.50 (t, J = 7.0 Hz, 3H). ^{13}C NMR (100 MHz, Chloroform-*d*) δ 159.4, 150.0, 148.5, 143.1, 134.5, 129.9, 129.0, 129.0, 128.3, 126.2, 126.1, 123.3, 120.1, 116.4, 114.6, 63.4, 14.6. HRMS (+ESI) m/z calcd. for $C_{19}H_{17}NO$ $(M+H)^+$ 276.1383, found 276.1382.

Synthesis of 1g

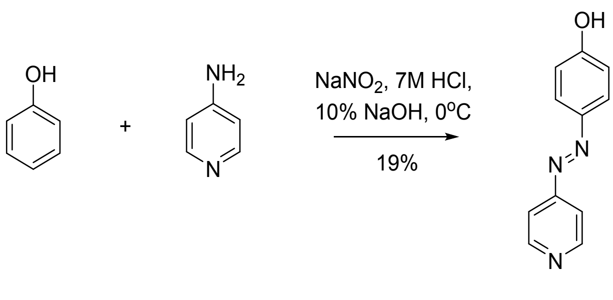

4-Aminopyridine was dissolved in 7M HCl (6.7 mL). A mixture containing 10% NaOH (3 mL), phenol (0.94g, 0.010 mol), and NaNO2 (0.768 g, 0.011 mol) was then added dropwise at 0 ℃ to

the solution of 4-Aminopyridine. The reaction was stirred for 2 hours. The product was precipitated and was isolated by filtration and crystallization from acetone/ ethanol to give a dark brow solid **1g** (384.8 mg, 19 % yield). mp = 254-255 ℃. ^{1}H NMR (400 MHz, DMSO-d_6) δ 10.64 (s, 1H), 9.37 – 8.32 (m, 2H), 7.89 (d, *J* = 8.8 Hz, 2H), 7.75 – 7.56 (m, 2H), 7.00 (d, *J* = 8.9 Hz, 2H). ^{13}C NMR (101 MHz, DMSO-d_6) δ 162.3, 156.8, 151.2, 145.2, 125.8, 116.2, 115.8. HRMS (+MALDI-TOF) m/z calcd. for $C_{11}H_9N_3O$ $(M+H)^+$ 200.0818, found 200.0816.

Synthesis of 1h

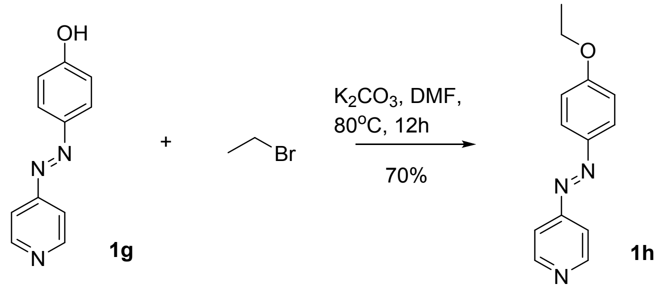

1g (99.8 mg, 0.5 mmol), ethyl bromide (0.1 mL, 1.5 mmol), K_2CO_3(208.6 mg, 1.5 mmol) was added into 5 mL of DMF. The resulting mixture was stirred at 80 °C for 12 h. The mixture was then diluted with water (20 mL), and the aqueous phase was extracted with ethyl acetate (15 mL × 3). The combined organic extracts were washed with water (15 mL × 3), dried over anhydrous Na_2SO_4, and evaporated under reduced pressure to give a dark brown solid (79.7mg, 70% yield) as the product **1h**. mp = 104 -105 ℃. ^{1}H NMR (400 MHz, chloroform-*d*) δ 8.70 (d, *J* = 5.8 Hz, 2H), 7.88 (dd, *J* = 9.1, 0.9 Hz, 2H), 7.60 (dd, J = 5.9, 1.1 Hz, 2H), 6.95 (d, J = 9.0 Hz, 2H), 4.07 (dd, *J* = 7.0, 1.2 Hz, 2H), 1.40 (td, *J* = 7.0, 1.0 Hz, 3H). ^{13}C NMR (101 MHz, Chloroform-*d*) δ 162.7, 157.5, 151.2, 146.7, 125.6, 116.2, 114.9, 64.0, 14.7. HRMS (+MALDI-TOF) m/z calcd. for $C_{13}H_{13}N_3O$ $(M+H)^+$ 228.1131, found 228.1144.

Synthesis of 1i

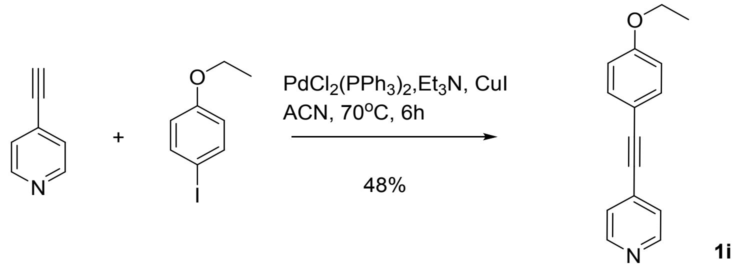

4-Iodophenetole (124.03 mg, 0.5 mmol), TEA (0.80 mL, 5.8 mmol), CuI (4.76 mg,5%), 5 mL ACN and a stir bar were placed in a two neck round bottom flask fitted with a reflux condenser. The bottom was degassed and protected by argon. Then palladium catalyst (35.1 mg, 10%) was quickly putted into the bottom. When the reaction was heated to 70 ℃, 4-ethynylpyridine dissolved in 5 mL ACN was slowly added into the reaction mixture. The reaction was stirred at reflux for 6 hours under the protection of argon. The mixture was diluted with water (20 mL), and extracted with EtOAc (15 mL×3), dried over anhydrous Na_2SO_4, and evaporated under reduced pressure. The reside was purified by flash column chromatography (EtOAc/ hexanes 1:1) to give a creamy solid as the product **1i** (106.3 mg, 48% yield). mp = 90-92 ℃. ^{1}H NMR (400 MHz, chloroform-*d*) δ 8.59 (s, 2H), 7.61 – 7.42 (m, 2H), 7.36 (d, J = 5.9 Hz, 2H), 6.95 – 6.80 (m, 2H), 4.07 (q, J = 7.0 Hz, 2H), 1.44 (t, J = 7.0 Hz, 3H). ^{13}C NMR (101 MHz, Chloroform-*d*) δ 159.7, 149.7, 133.4, 131.8, 125.4, 114.6, 113.9, 94.4, 85.6, 63.6, 14.7. HRMS (+ESI) m/z calcd. for $C_{15}H_{13}NO$ $(M+H)^+$ 223.0997, found 223.1071.

Synthesis of 1j

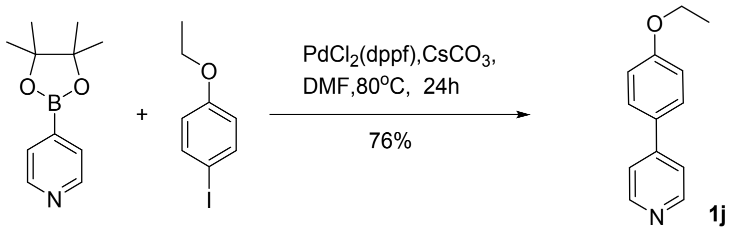

4-Iodophenetole (124.0 mg, 0.5 mmol), 4-Pyridineboronic acid pinacol ester (102.5 mg, 0.5 mmol), Cs_2CO_3 (814.6 mg, 2.5 mmol), 10 mL DMF and a stir bar were placed in a two neck round bottom degassed and charged with argon. Then the palladium catalyst (40 mg, 10%) was quickly putted into the bottom. The bottom was fitted with a reflux condenser and then charged again with argon.

The reaction was heated at 100 ℃ for 24 hours. The mixture was diluted with water (20 mL), and extracted with EtOAc (15 mL×3), dried over anhydrous Na_2SO_4, and evaporated under reduced pressure. The reside was purified by flash column chromatography (EtOAc/ hexanes 3:1) to get a creamy solid as the product **1j** (76 mg, 76% yield). Mp = 100-101℃. ^{1}H NMR (400 MHz, chloroform-*d*) δ 8.61 (d, *J* = 4.2 Hz, 2H), 7.59 (d, *J* = 8.6 Hz, 2H), 7.47 (d, *J* = 5.9 Hz, 2H), 7.00 (d, *J* = 8.7 Hz, 2H), 4.09 (q, *J* = 7.0 Hz, 2H), 1.45 (t, *J* = 7.0 Hz, 3H). ^{13}C NMR (101 MHz, Chloroform-*d*) δ 159.9, 150.2, 147.8, 130.1, 128.1, 121.0, 115.0, 63.6, 14.8. HRMS (+ESI) m/z calcd. for $C_{13}H_{13}NO$ $(M+H)^+$ 200.0997, found 200.1071.

2. NMR spectra of new compounds

^{1}H NMR and ^{13}C NMR spectra of **1a** in $CDCl_3$

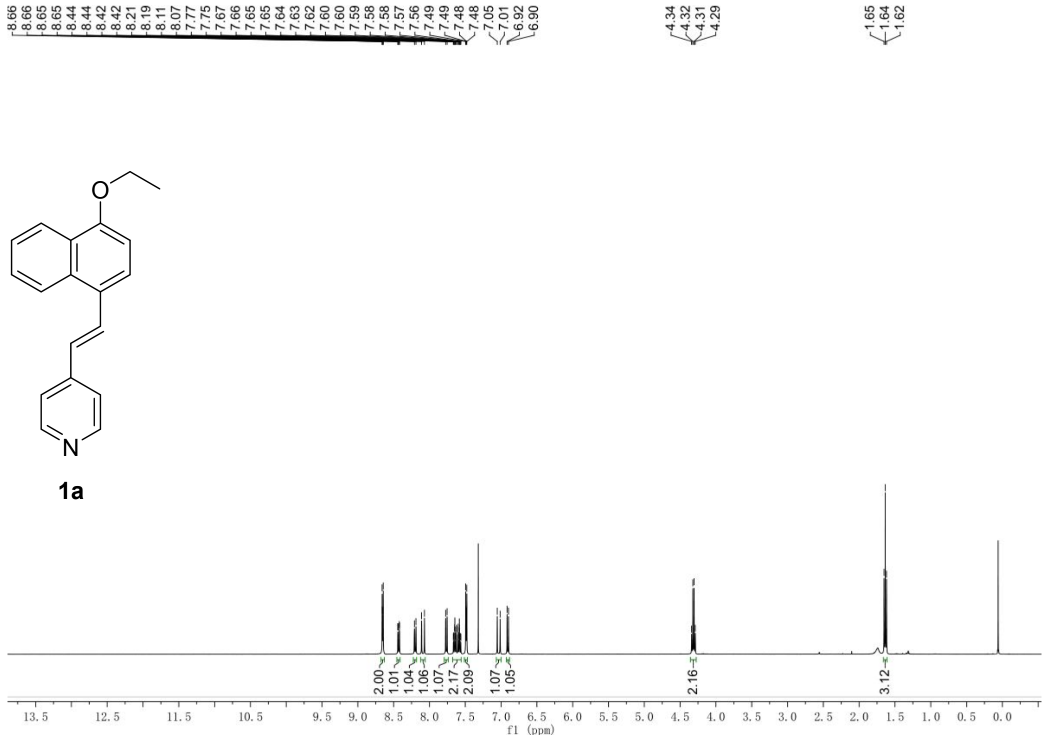

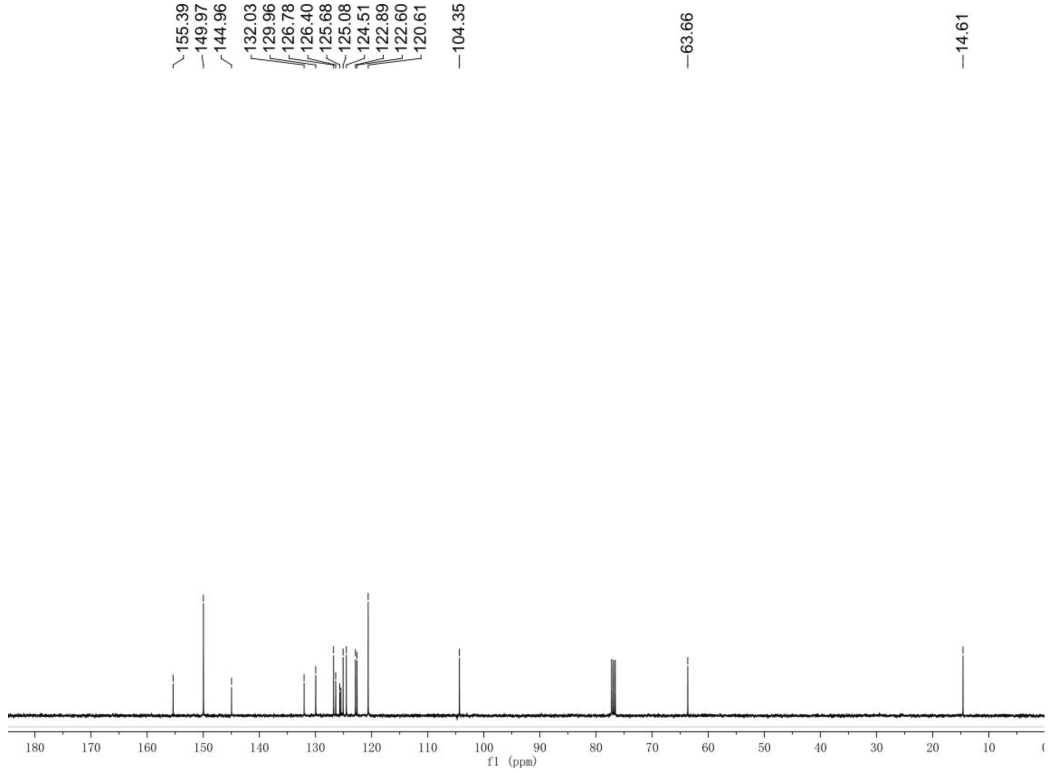

^{1}H NMR and ^{13}C NMR spectra of **1b** in $CDCl_3$

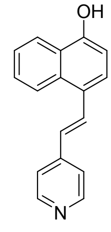

1b

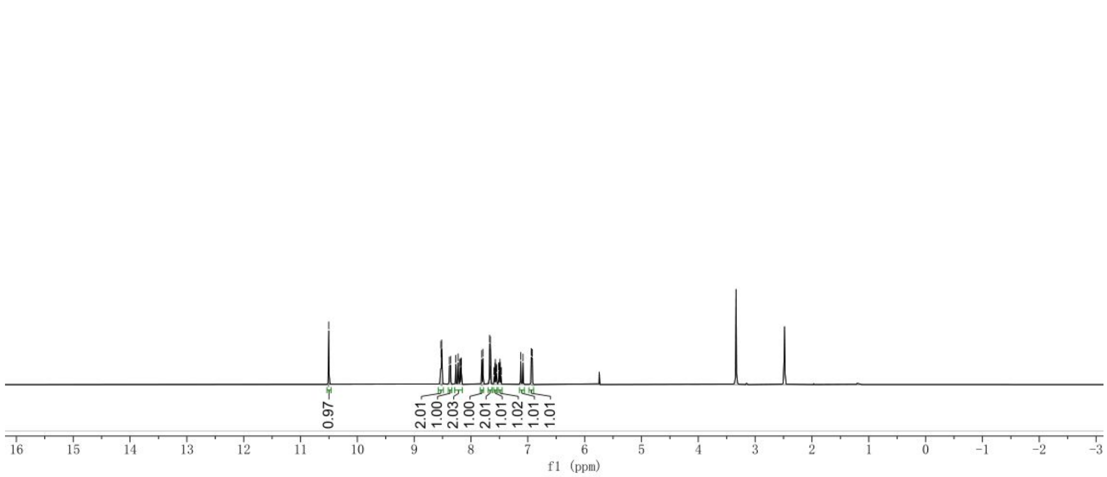

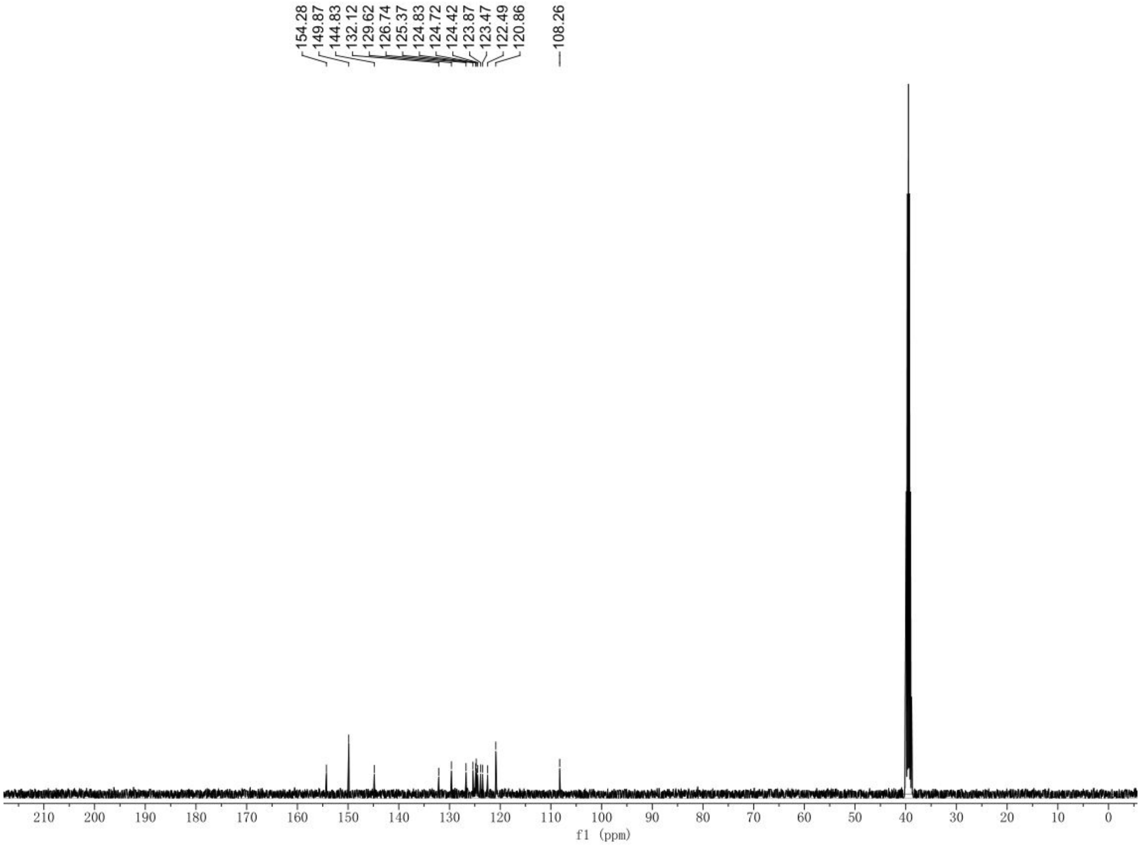

^{1}H NMR and ^{13}C NMR spectra of **1c** in DMSO-d_6

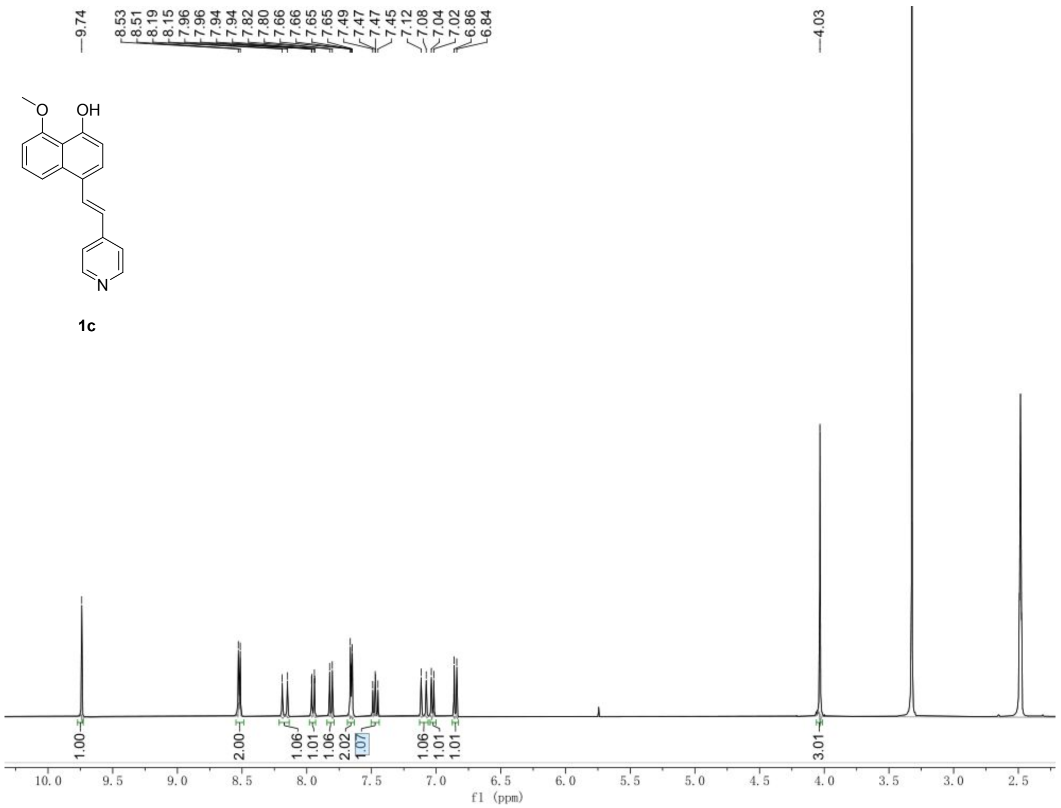

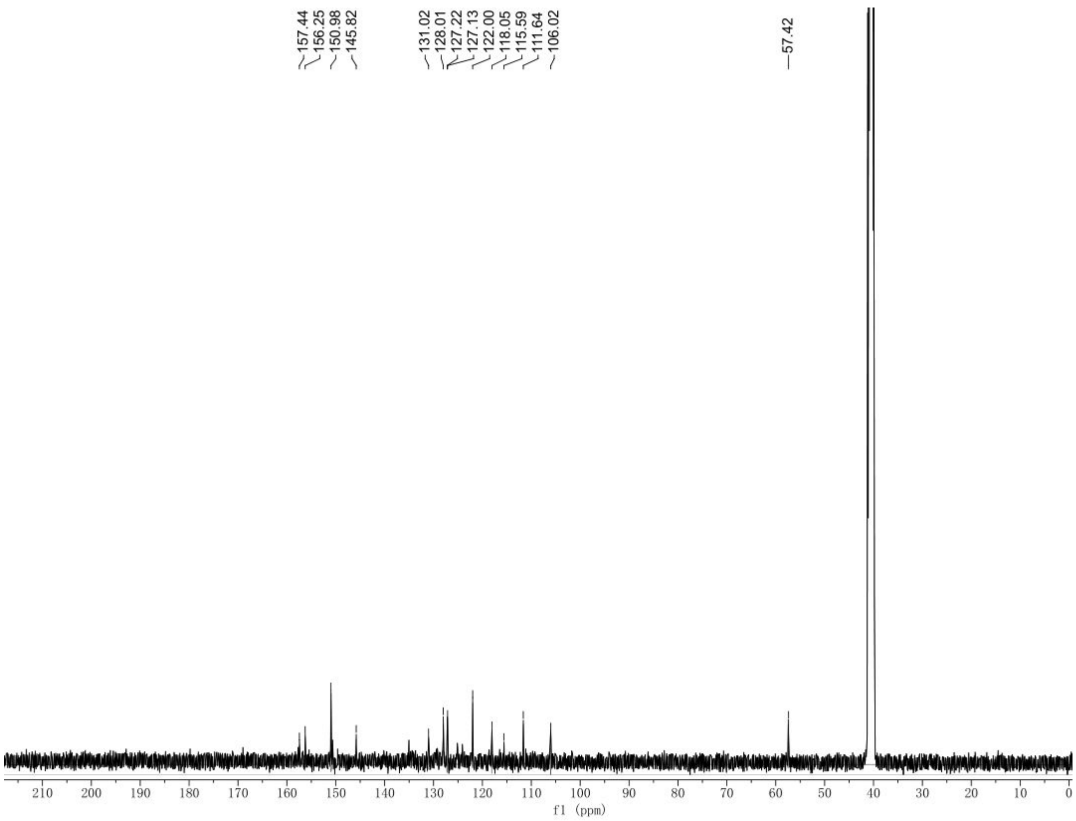

^{1}H NMR and ^{13}C NMR spectra of **1d** in $CDCl_3$

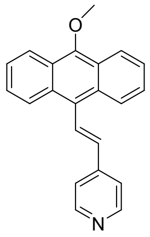

1d

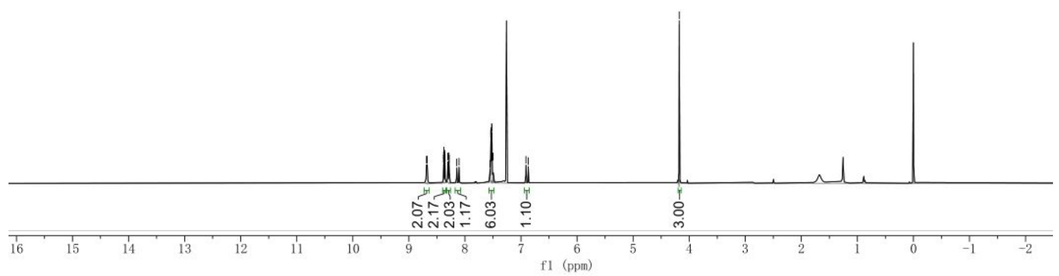

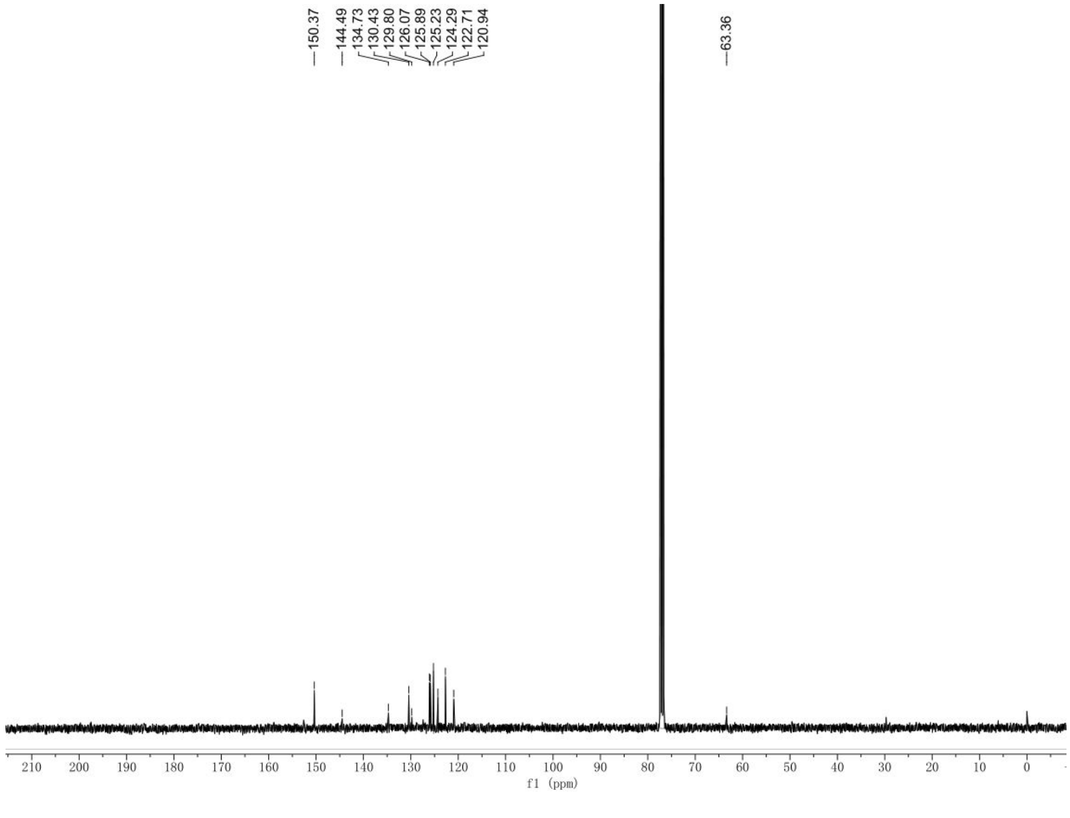

^{1}H NMR and ^{13}C NMR spectra of **1e** in $CDCl_3$

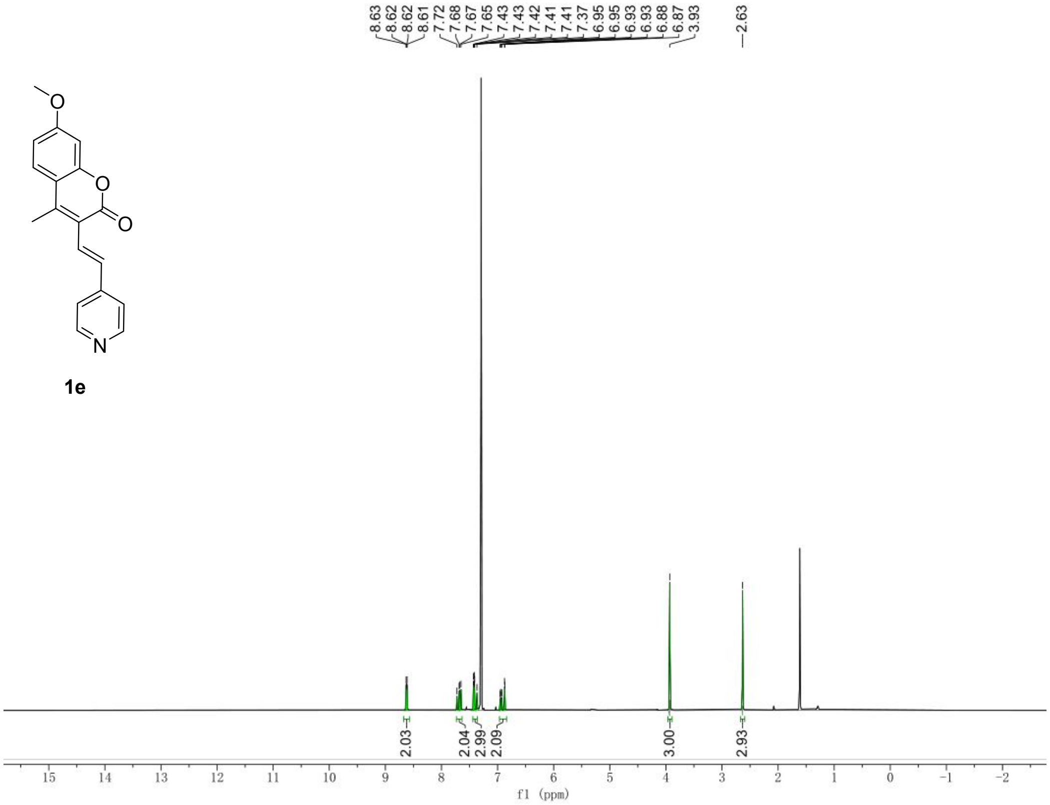

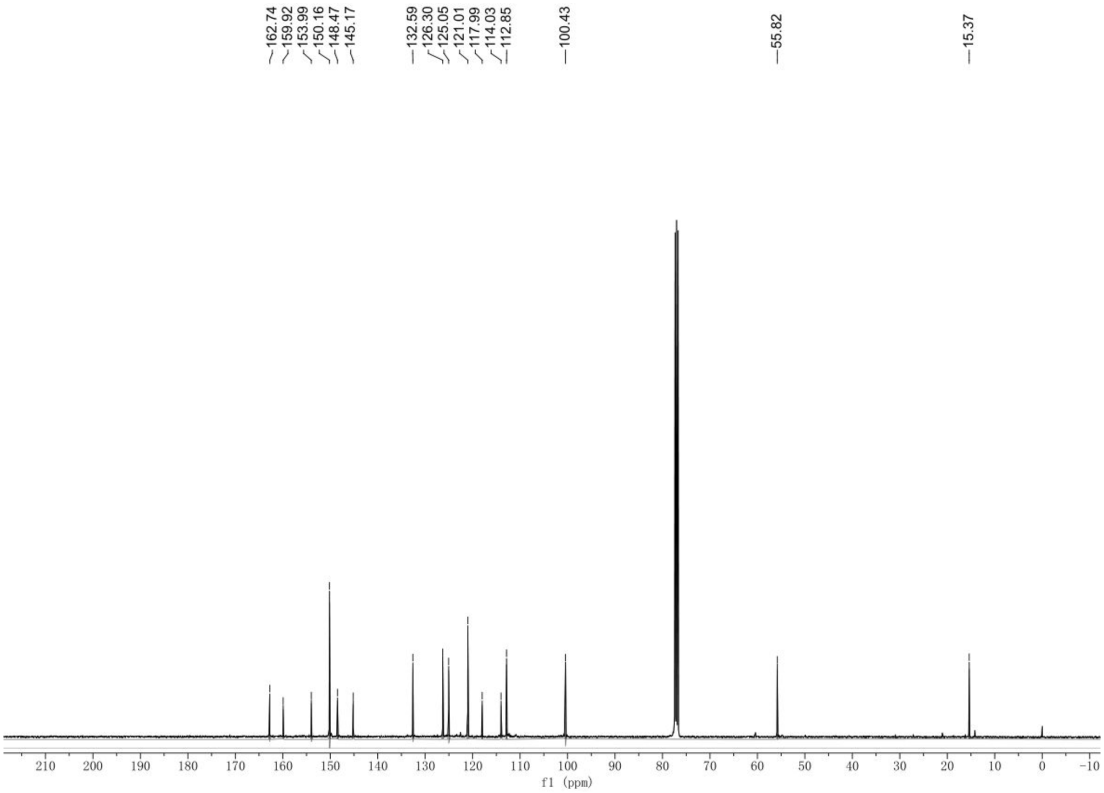

^{1}H NMR and ^{13}C NMR spectra of **1f** in $CDCl_3$

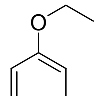

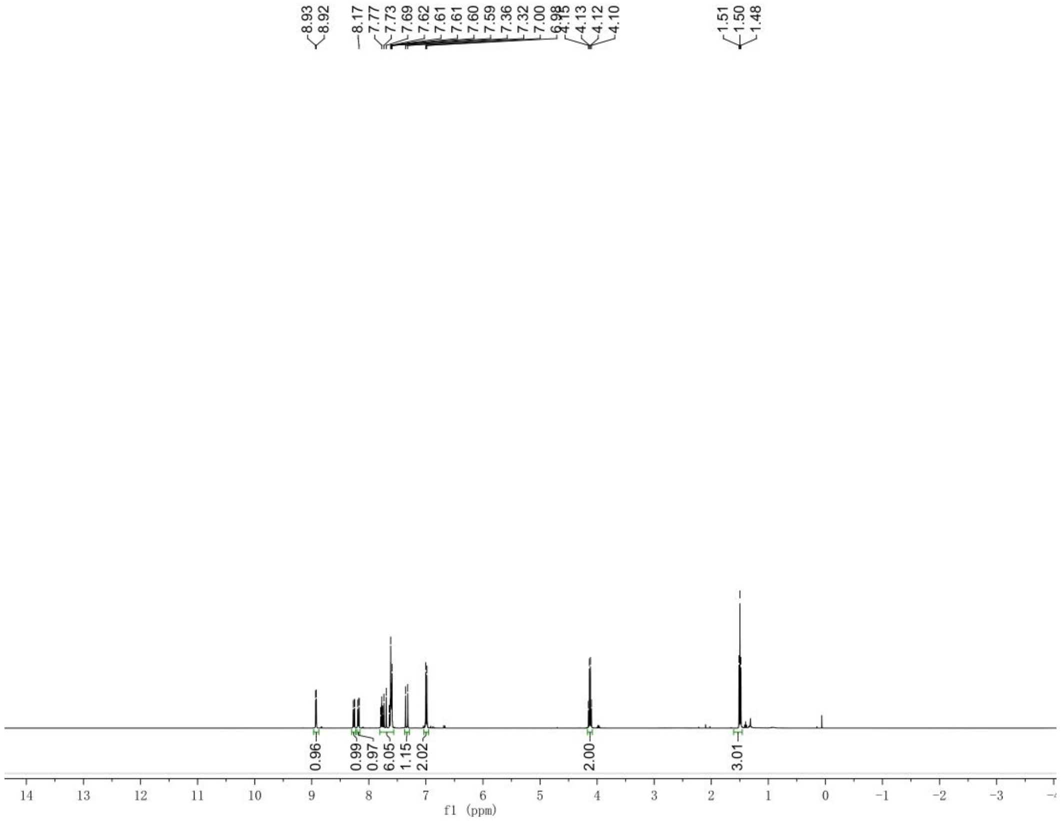

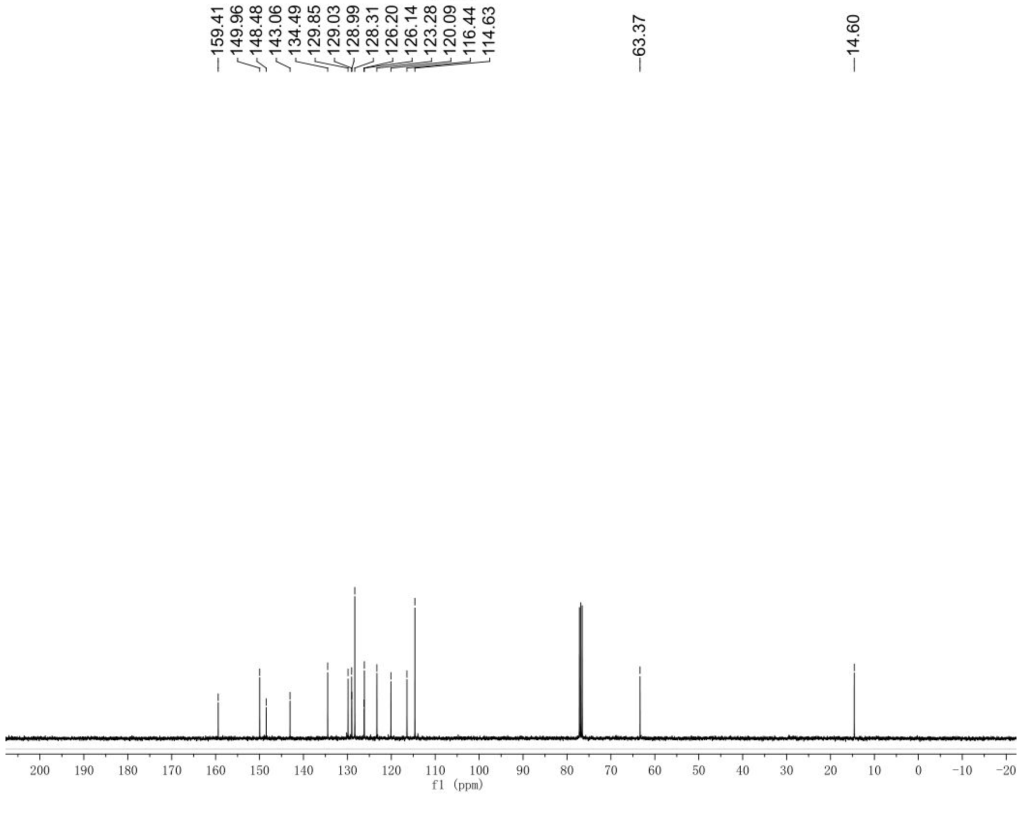

^{1}H NMR and ^{13}C NMR spectra of **1g** in DMSO-d_6

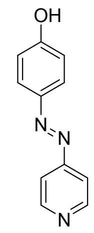

1g

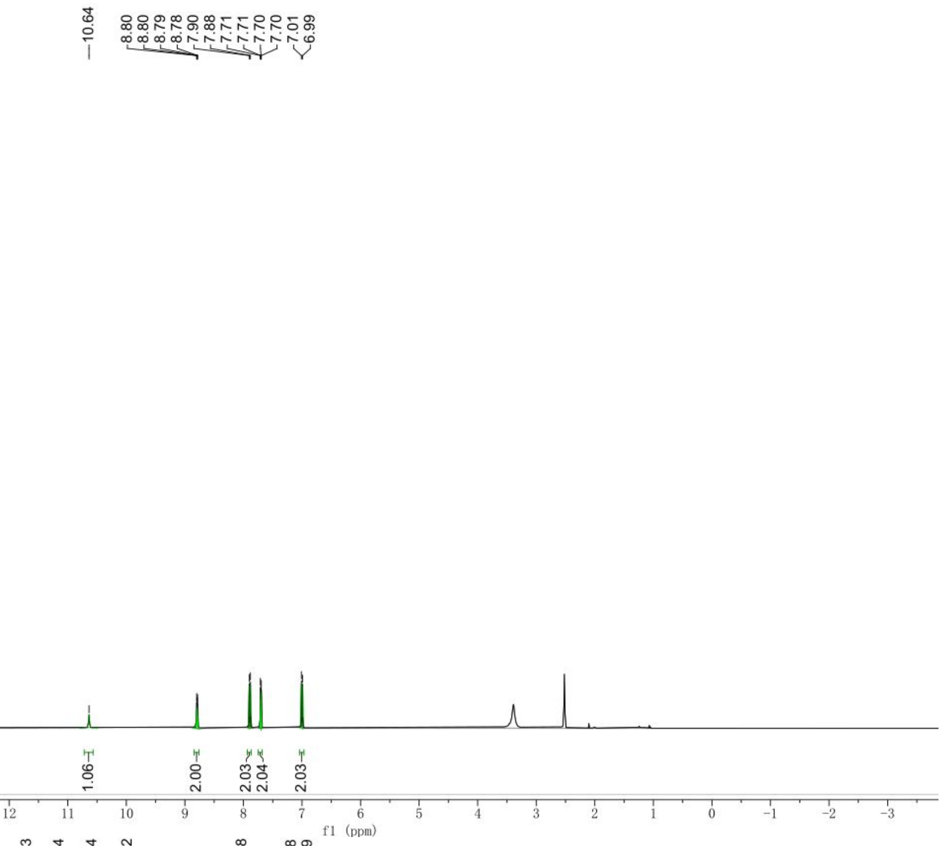

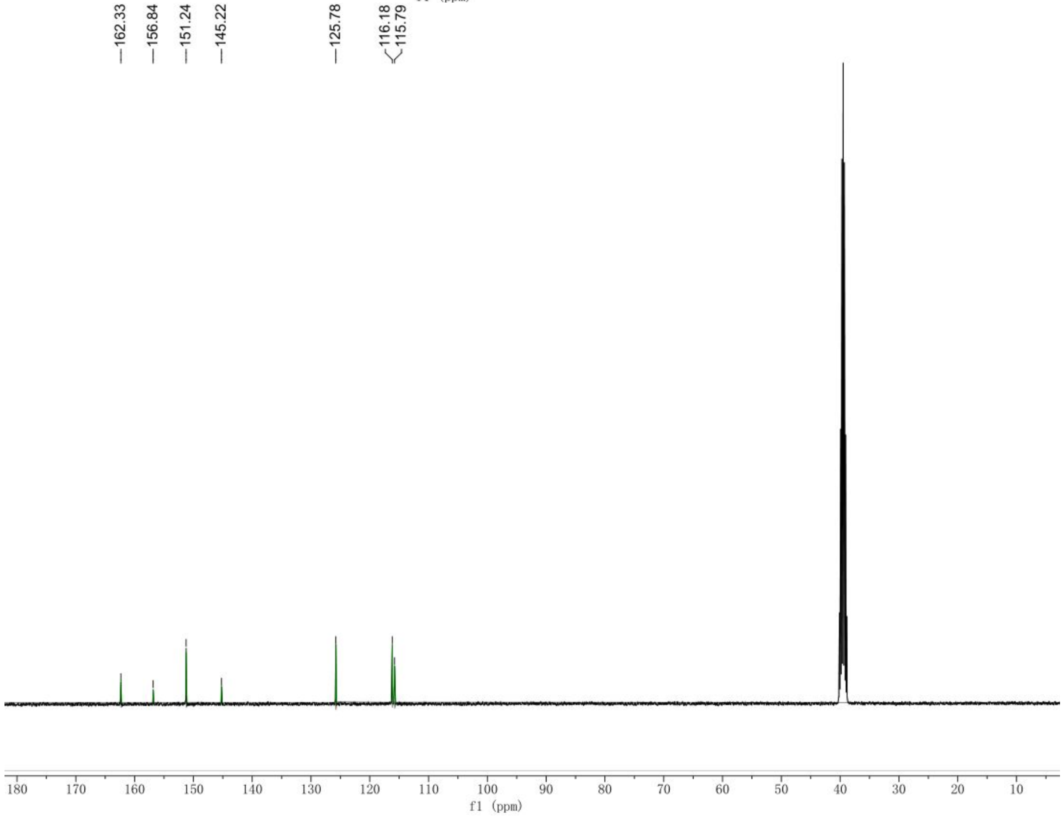

^{1}H NMR and ^{13}C NMR spectra of **1h** in $CDCl_3$

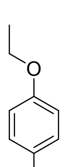

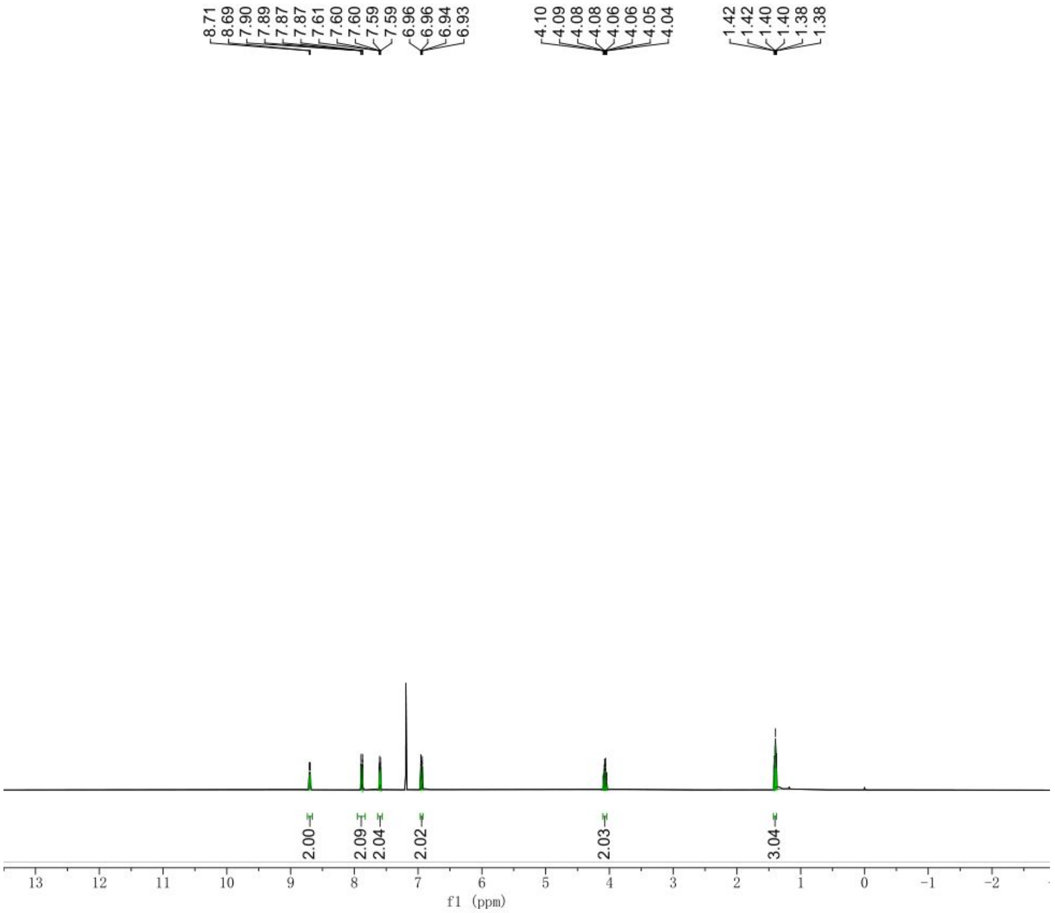

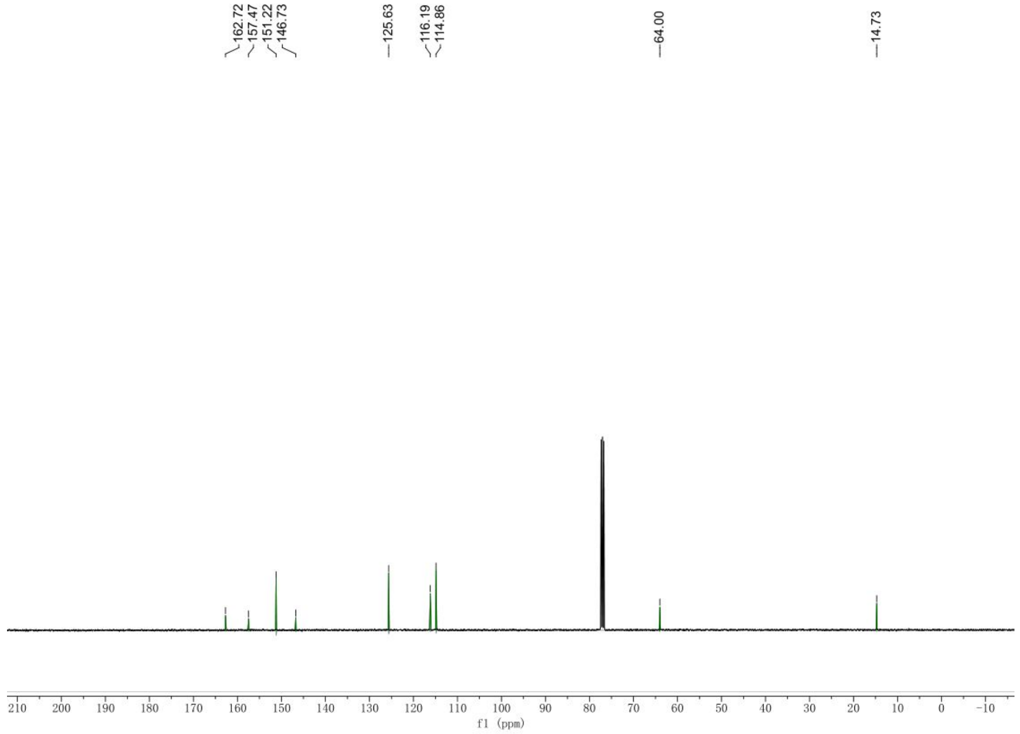

^{1}H NMR and ^{13}C NMR spectra of **1i** in $CDCl_3$

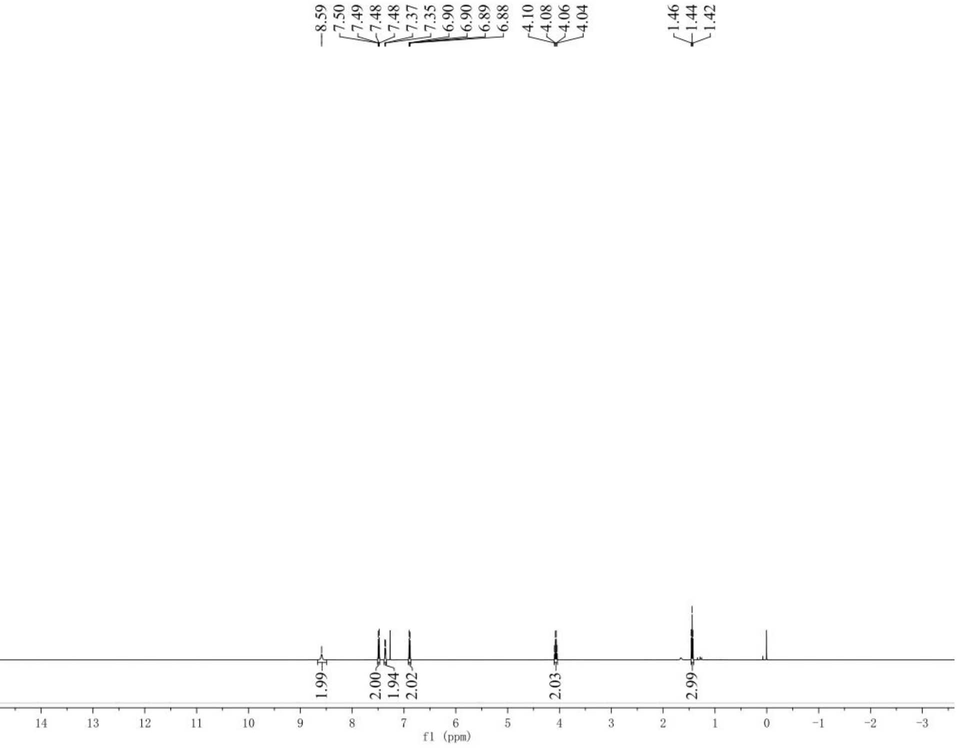

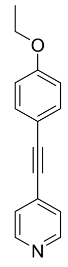

1i

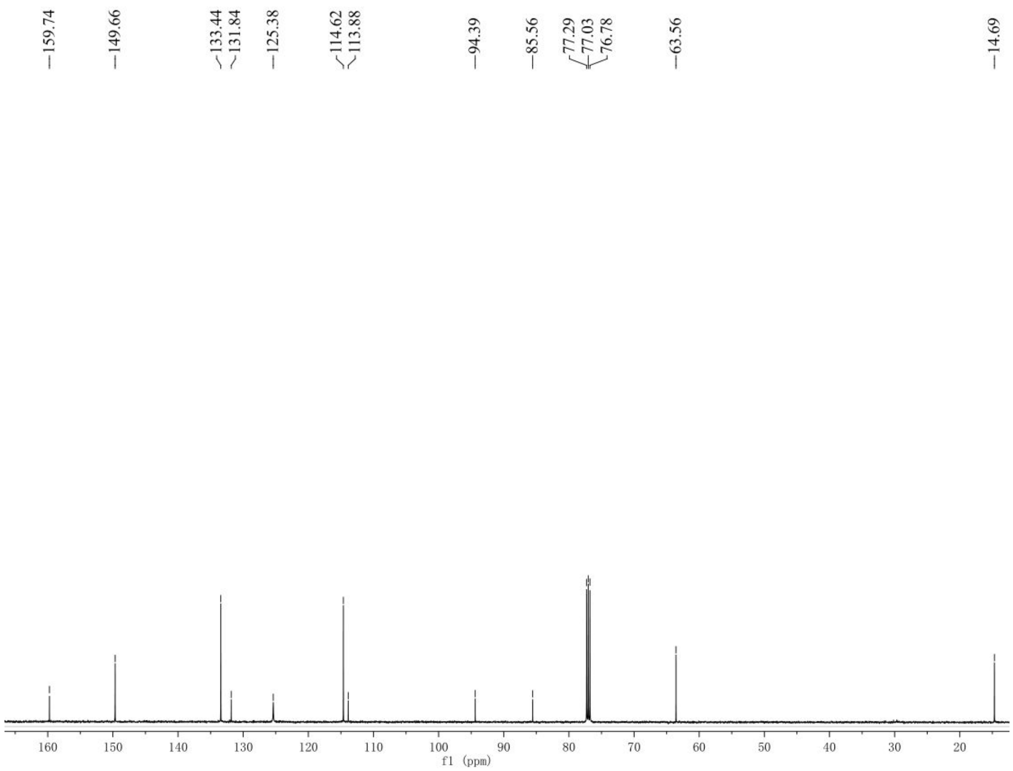

^{1}H NMR and ^{13}C NMR spectra of **1j** in $CDCl_3$

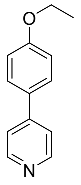
O
N

1j

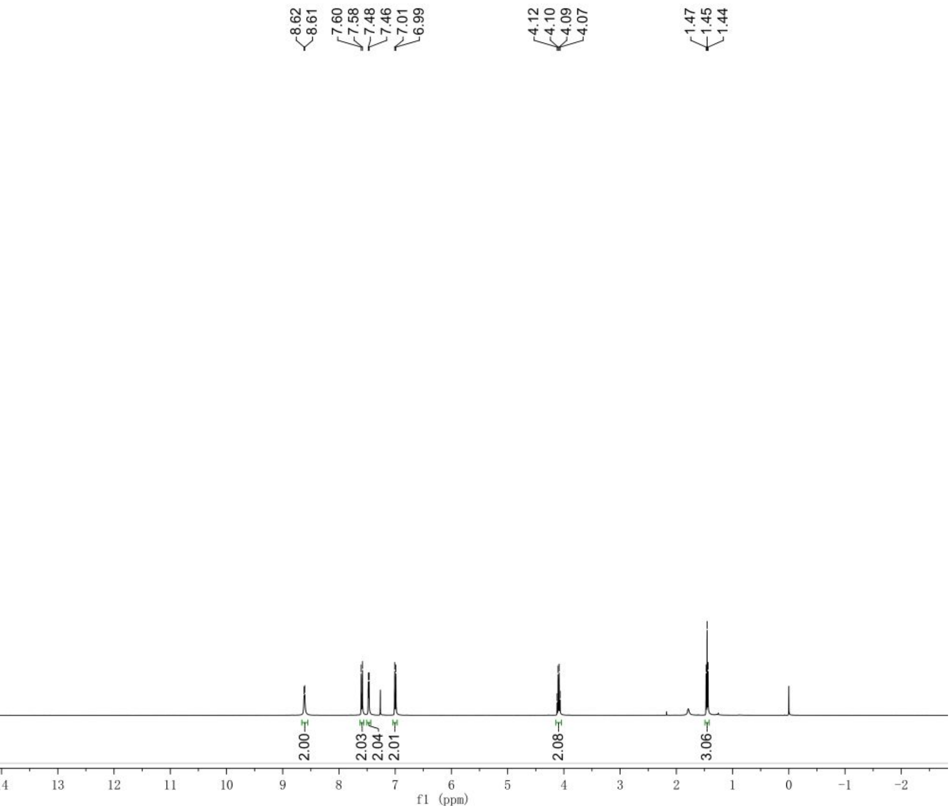
8.62
8.61
7.60
7.58
7.48
7.46
7.01
6.99
4.12
4.10
4.09
4.07
1.47
1.45
1.44
2.00
2.03
2.04
2.01
2.08
3.06
14 13 12 11 10 9 8 7 6 5 4 3 2 1 0 -1 -2
f1 (ppm)

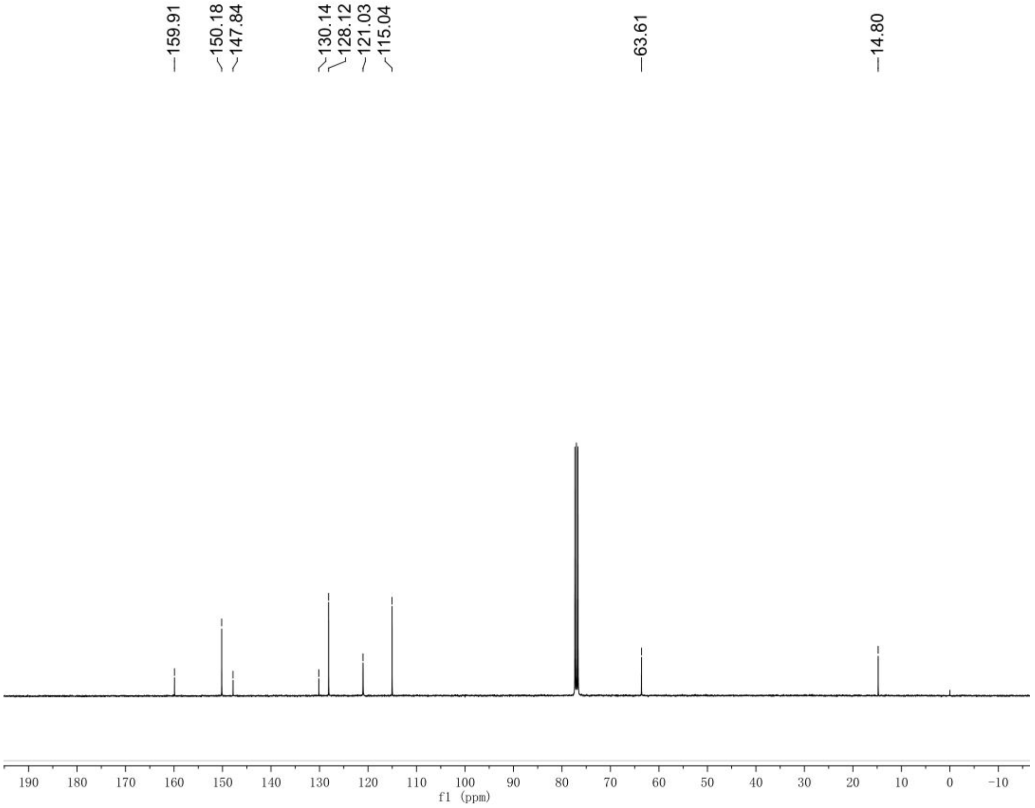
159.91
150.18
147.84
130.14
128.12
121.03
115.04
63.61
14.80
190 180 170 160 150 140 130 120 110 100 90 80 70 60 50 40 30 20 10 0 -10
f1 (ppm)

3. Supplementary Figures and Tables

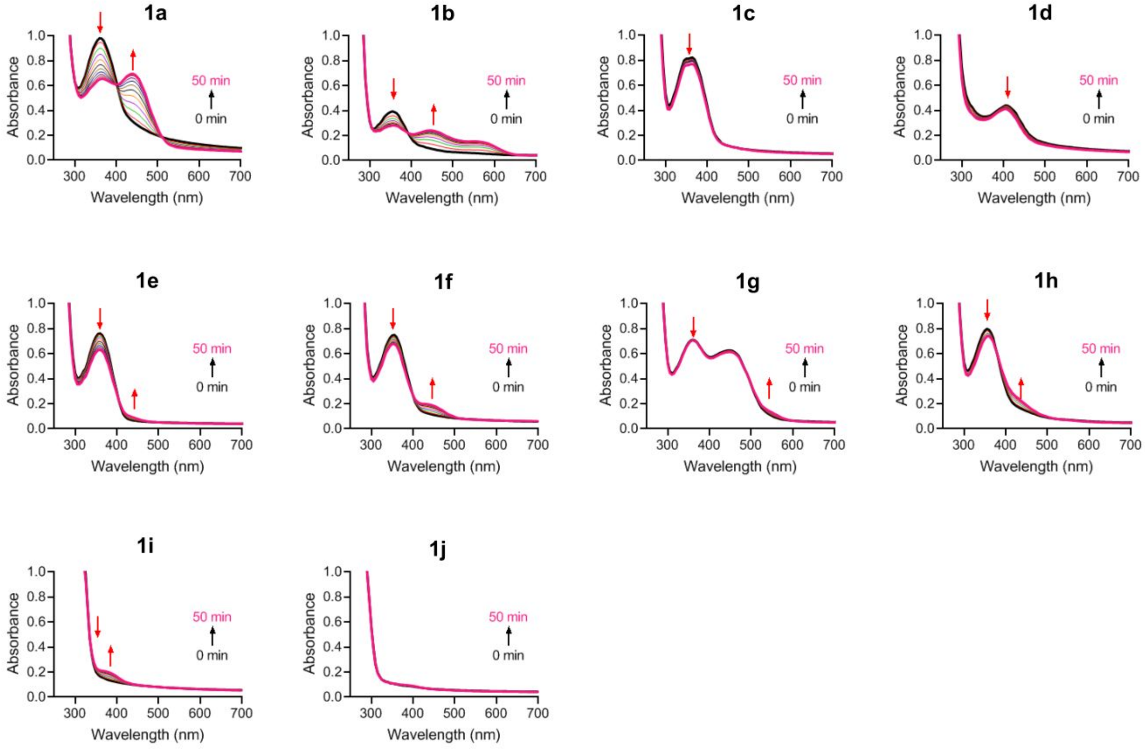

Figure S1. UV absorbance changes of the base-exchange reactions of **1a** – **1j** catalysed by activated SARM1-dN

Table S1. UV-vis absorption changes of **1a** – **1j** in the base-exchanged reactions

Probe	λ_{max} (nm)	λ_{new} (nm)	Isosbestic point (nm)
**1a**	360	445	380
**1b**	355	455, 590	390
**1c**	361	-	-
**1d**	404, 461	-	-
**1e**	358	430	400
**1f**	364	440	390
**1g**	368, 441	550	500
**1h**	354	415	380
**1i**	308	380	320
**1j**	284	-	-

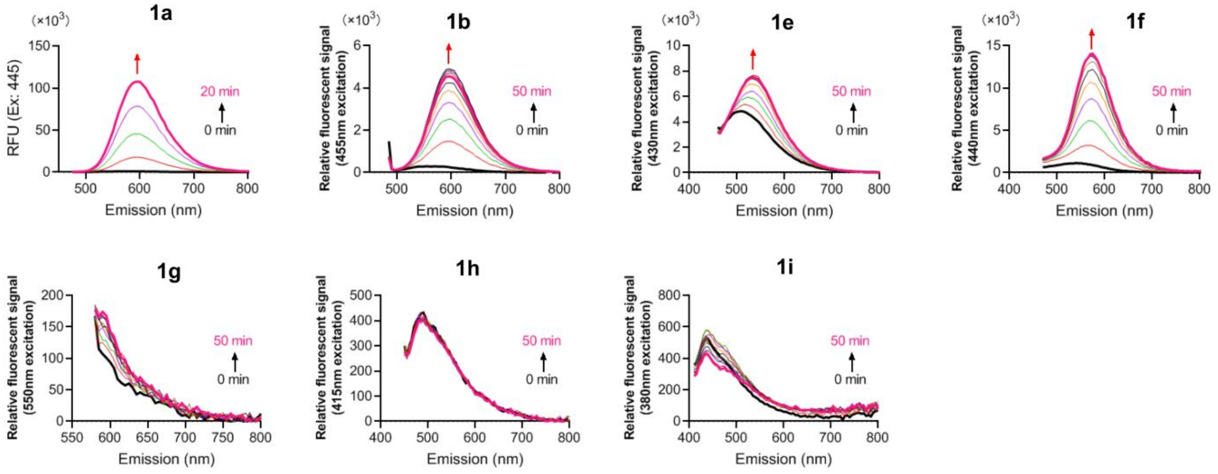

Figure S2. Florescence changes of the exchange reactions of **1a**, **1b** and **1e** – **1i** catalyzed by activated SARM1-dN

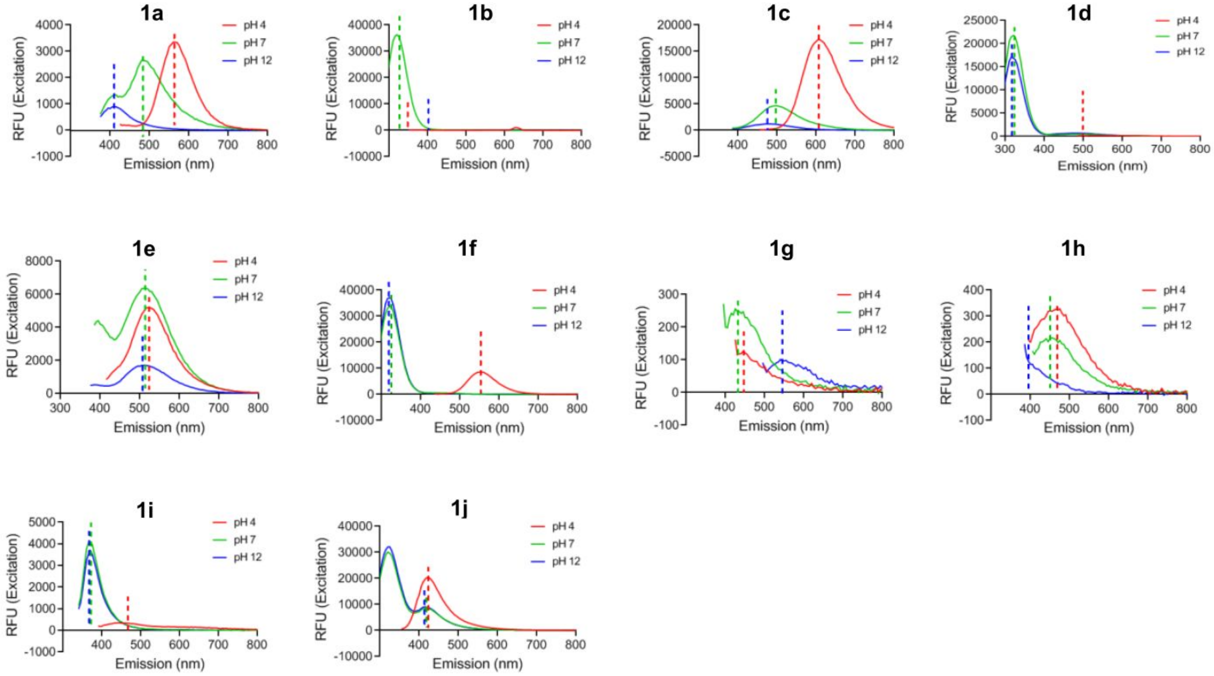

Figure S3. Fluorescent changes of **1a** – **1j** at different pH

Table S2. Fluorescence properties of probe **1a**, **1b**, **1e**, **1f** and their base-exchanged products

Probe / Base-exchanged product	λ_{ex} (nm)	λ_{em} (nm)	Redshift (nm)	Stokes shift (nm)	Fluorescence enhancement rate (RFU/min)
**1a** / AD-**1a**	360 / 445	485 / 595	110	150	4720 ± 290
**1b** / AD-**1b**	355 / 455	490 / 590	100	135	264 ± 20
**1e** / AD-**1e**	358 / 430	483 / 530	47	100	135 ± 15
**1f** / AD-**1f**	364 / 440	457 / 580	123	140	528 ± 8

Reference

1. Fan, Y.; Feng, P.; Liu, M.; Pan, H.; Shi, Y. A concise approach to the dalesconol skeleton. *Org. Lett.* **2011**, *13*, 4494-4497.
2. Hirao, Y. Saito, T. Kurata, H. Kubo,T. Isolation of a hydrogen-bonded complex based on the anthranol/anthroxyl pair: formation of a hydrogen-atom self-exchange system. *Angew. Chem. Int. Ed.* **2015**, *54*, 2402-2405.
3. Li, W. H. Huang, K. Cai, Y. Wang, Q. W. Zhu, W. J. Hou, Y. N. Wang, S. Cao,S. Zhao, Z. Y. Xie, X. J. Du, Y. Lee, C.-S. Lee, H. C. Zhang, H. Zhao, Y. J. Permeant fluorescent probes visualize the activation of SARM1 and uncover an anti-neurodegenerative drug candidate. *eLife* **2021**, *10*, e67381.
